# Transcriptome analysis of *Plasmodium berghei* during exo-erythrocytic development

**DOI:** 10.1186/s12936-019-2968-7

**Published:** 2019-09-24

**Authors:** Reto Caldelari, Sunil Dogga, Marc W. Schmid, Blandine Franke-Fayard, Chris J. Janse, Dominique Soldati-Favre, Volker Heussler

**Affiliations:** 1 Institute of Cell Biology, University of Bern, Bern, Switzerland; 2 Department of Microbiology and Molecular Medicine, Faculty of Medicine, University of Geneva CMU, Geneva, Switzerland; 3 MWSchmid GmbH, Zurich, Switzerland; 4 Leiden Malaria Research Group, Department of Parasitology, Leiden University Medical Center, Leiden, The Netherlands

**Keywords:** Malaria, Life cycle, *Plasmodium* liver stage, Transcriptome, RNA-seq, Merosome

## Abstract

**Background:**

The complex life cycle of malaria parasites requires well-orchestrated stage specific gene expression. In the vertebrate host the parasites grow and multiply by schizogony in two different environments: within erythrocytes and within hepatocytes. Whereas erythrocytic parasites are well-studied in this respect, relatively little is known about the exo-erythrocytic stages.

**Methods:**

In an attempt to fill this gap, genome wide RNA-seq analyses of various exo-erythrocytic stages of *Plasmodium berghei* including sporozoites, samples from a time-course of liver stage development and detached cells were performed. These latter contain infectious merozoites and represent the final step in exo-erythrocytic development.

**Results:**

The analysis represents the complete transcriptome of the entire life cycle of *P. berghei* parasites with temporal detailed analysis of the liver stage allowing comparison of gene expression across the progression of the life cycle. These RNA-seq data from different developmental stages were used to cluster genes with similar expression profiles, in order to infer their functions. A comparison with published data from other parasite stages confirmed stage-specific gene expression and revealed numerous genes that are expressed differentially in blood and exo-erythrocytic stages. One of the most exo-erythrocytic stage-specific genes was PBANKA_1003900, which has previously been annotated as a “gametocyte specific protein”. The promoter of this gene drove high GFP expression in exo-erythrocytic stages, confirming its expression profile seen by RNA-seq.

**Conclusions:**

The comparative analysis of the genome wide mRNA expression profiles of erythrocytic and different exo-erythrocytic stages could be used to improve the understanding of gene regulation in *Plasmodium* parasites and can be used to model exo-erythrocytic stage metabolic networks toward the identification of differences in metabolic processes during schizogony in erythrocytes and hepatocytes.

## Background

Malaria is a devastating disease caused by apicomplexan parasites of the genus *Plasmodium*. Almost half of the world’s population is permanently at risk of malaria resulting in over 200 Million malaria cases worldwide mostly in African countries. There were more than 400,000 deaths in 2017 [1], and the majority of them were children under the age of five.

The life cycle of *Plasmodium* parasites involves the injection of sporozoites into the vertebrate host during a blood meal of an infected female mosquito. For the rodent parasite *Plasmodium berghei* it has been shown that a proportion of injected sporozoites actively invade blood vessels and then are passively transported to the liver [2]. After crossing the blood vessel endothelia in the liver to reach the parenchyma, the parasite transmigrates through several hepatocytes before it settles in one. Upon entry into the ultimate host cell, the host plasma membrane invaginates forming a parasitophorous vacuole (PV) in which the parasite resides, develops and multiplies by exo-erythrocytic schizogony. The intracellular parasite extensively remodels the parasitophorous vacuole membrane (PVM), in particular by excluding or removing host cell proteins and by incorporating parasite proteins [3].

Each exo-erythrocytic stage parasite (EEF: exo-erythrocytic form) generates tens of thousands of nuclei by the process of exo-erythrocytic schizogony. This rapid nuclear division is accompanied by growth and replication of organelles including the Golgi apparatus, endoplasmic reticulum, mitochondrion and apicoplast and a vast expansion of the plasma membrane [4–6]. Nuclei and organelles are eventually segregated into individual merozoites. Once EEF merozoites have completed their development, the PVM ruptures. This process requires an orchestrated action of multiple *Plasmodium* proteins such as lipases (e.g. PbPL) [7], proteases (e.g. SUB1) [8, 9] and possibly perforins as shown for erythrocytic stage parasites (EF: erythrocytic form) [10–12]. Upon rupture of the PVM, EEF merozoites disperse in the host cell cytoplasm and the host cell actin cytoskeleton collapses [13]. The final developmental stage of the EEF in in vitro cultures, is the formation of detached cells (DCs) and merosomes, host cell plasma membrane enclosed merozoites [14, 15]. In the in vivo mouse model, infected cells become excluded from the liver tissue upon PVM rupture and merosomes are formed and pushed into the lumen of adjacent blood vessels. At tissue sites with small capillaries, merosomes rupture to release merozoites into the blood [16]. Liberated merozoites immediately invade red blood cells (RBC), where they undergo repeated asexual reproduction cycles. In contrast to the tens of thousands merozoites generated by a single EEF, within erythrocytes the parasites produce only a limited number (12 to 32) merozoites by erythrocytic schizogony. Another difference between erythrocytic and exo-erythrocytic schizogony is that after rupture of the PVM the EEF merozoites can reside in the host cell cytoplasm for up to several hours, whereas EF merozoites are liberated from the PVM and the host cell plasma membrane almost simultaneously [17]. Some EF will differentiate into male and female gametocytes. When these are ingested by a mosquito during a blood meal, they mature into macrogametes and microgametes and are liberated from the RBC. These sexual forms fuse to form zygotes and transform into motile ookinetes, which are able to cross the midgut epithelium of the mosquito to develop into oocysts, in which thousands of sporozoites are formed. After about 9–16 days (depending on the parasite species and the environmental temperature), sporozoites are liberated and invade the salivary glands of the mosquito (reviewed in [18]), whereupon they are then ready to be injected into a host during the next blood meal.

Studying the entire life cycle using human parasites under live or laboratory conditions is difficult due to ethical and safety reasons. However, the use of model organisms, such as the rodent malaria parasite *P. berghei* is experimentally tractable and the genetic manipulation of this parasite is relatively easy and well established [19–21]. This model allows investigating the EEF development and filling gaps of knowledge that might also be relevant for human *Plasmodium* species. Many transcriptomic studies have been undertaken for different *Plasmodium* species, either by microarrays or RNA-seq. Table 1 lists the transcriptomic data integrated in PlasmoDB [22].

**Table 1:** Genome wide transcriptome data from studies integrated in PlasmoDB release43; http://www.plasmodb.org (np: not published)

Method	*Plasmodium* sp.	Life cycle stages	Refs.
RNA-seq	*P. berghei* ANKA	5 asexual and sexual stage transcriptomes	[49]
		Female and male gametocyte	[85]
	*P. chabaudi chabaudi*	Trophozoite transcriptomes after mosquito transmission or direct injection into mice	[86]
	*P. yoelii yoelii* 17X	Salivary gland sporozoite transcriptomes: WT vs. Puf2-KO	[87]
	*P. falciparum* 3D7	NSR-seq transcript profiling of malaria-infected pregnant women and children	[88]
		Polysomal and steady-*P. berghei* state asexual stage transcriptomes	[89]
		Blood stage transcriptome (3D7)	[90]
		Transcriptomes of 7 sexual and asexual life stages	[91]
		Intraerythrocytic cycle transcriptome (3D7)	np
		Strand specific transcriptomes of 4 life cycle stages	[91]
		Transcriptome during intraerythrocytic development	[92]
		Strand specific transcriptome of the intraerythrocytic developmental cycle	[93]
		Ribosome and steady state mRNA sequencing of asexual cell cycle stages	[94]
		Mosquito or cultured sporozoites and blood stage transcriptome (NF54)	np
		Female and male gametocyte transcriptomes	[50]
		Ring, oocyst and sporozoite transcriptomes	[95]
	*P. vivax* P01	Hypnozoite RNAseq	[29]
		Transcription profile of intraerythrocytic cycle	[96]
	*P. cynomolgi* strain M	*P. cynomolg*i transcriptome of whole blood and bone marrow collected during 100-day infection of *M. mulatta*	[97]
		Hypnozoite, schizont and blood stage transcriptomes (laser microdissection)	[98]
		Liver stage hypnozoite vs schizont transcriptomes (primary culture)	[30]
DNA micro-array	*P. berghei* ANKA	DOZI mutant transcript profile	[51]
		Transcript profiling of developmental stages—high producer (HP/HPE)	[99]
		AP2-G2 knock out and WT expression profiles	[100]
	*P. yoelii yoelii* 17X	Liver, mosquito and blood stage expression profiles	[28]
		Life cycle stages	[101]
	*P. falciparum* 3D7	Pfal3D7 real-time transcription and decay	np
		Invasion pathway knockouts	[102]
		Three isogenic lines w/CQ treatment: expression profiles	[103]
		Life cycle expression data (3D7)	[104]
		Sexually vs asexually committed schizont transcriptional profiles	[105]
		Gametocyte stages I-V transcriptomes	[106]
		Two Sir2 KO lines expression profiling	[107]
		Asexual blood stage transcriptomes of clonal strains	[108]
		Erythrocytic expression time series (3D7, DD2, HB3)	[109, 110]
		Microarray expression from patient samples	[111]
		mRNA half life	[112]
	*P. vivax* P01	Intraerythrocytic developmental cycle of three isolates	[113]
		Sporozoite expression profiles	[114]
	*P. knowlesi* strain H	Intraerythrocytic cycle expression profile: in vitro and ex vivo (Pkno PK1(A+))	[115]

Recently, single cell transcriptomic profiles have also been published for EF of *Plasmodium falciparum, Plasmodium knowlesi* and *P. berghei* [23–25]. One of these studies (the Malaria Cell Atlas) also analysed the liver stage, but only a single time point (44hpi) [25]. Two further RNA-seq studies focused on the host cell transcriptome and did not attempt to group parasite genes in a systematic manner and did not include data from later stage EEFs (beyond 48hpi) [26, 27]. Three further studies analysed the transcriptome of *Plasmodium* EEFs, one of which was done using the rodent parasite *Plasmodium yoelii* [28] and the other two using *Plasmodium vivax* with an emphasis on the dormant parasite stage, the hypnozoite [29, 30]. Since *P. berghei* is a widely used model in malaria research and the so far published data for its EEF stages are incomplete, genome wide RNA-seq analyses of EEF development in a time course fashion were performed and expression data were compared to already published data of gene-expression of other life cycle stages of *P. berghei*. In particular, EEF merozoites originating from DCs/merosomes were compared to EF merozoites (from in vitro cultivated schizonts) and it was found that their transcription profiles differ substantially. Remarkably, differences were identified that are predicted to have an impact on metabolic processes during schizogony in erythrocytes and hepatocytes despite the fact, that both types of merozoites infect RBC.

## Methods

### Mice, parasites, infections

BALB/c mice used for mosquito infections were between 6 and 10 weeks of age and were purchased from the Central animal facility at University of Bern, Harlan (Horst, the Netherlands) or Janvier Labs (Le Genest Saint Isle, France). For RNA work: mice were infected by intraperitoneal injection of blood stabilates of marker-free *P. berghei* strain ANKA expressing mCherry under the control of hsp70 regulatory sequences (PbmCherry_hsp70_) [7]. At parasitaemia of ~ 4%, the mouse was bled and 40 μl of infected blood was injected intravenously into phenylhydrazine treated mice (200 μl of 6 mg/ml in PBS, 2–3 days before). At day 3 to 4 after infection, each mouse with at least 7% parasitaemia was anaesthetized with Ketasol/Xylasol and exposed to ~ 150 female *Anopheles stephensi* mosquitoes (which were sugar starved for 5 h). Mosquitoes were kept at 20.5 °C and 80% humidity. From day 16–26 post infection salivary glands of infected mosquitos (sorted by fluorescence stereomicroscope *Olympus SZX10/U*-*HGLGPS*) were dissected into serum free IMDM (Iscove’s Modified Dulbecco’s Medium, Sigma-Aldrich). Sporozoites were liberated from the glands and were used to infect confluent HeLa cells (per time point 10 wells of 96 well plates were seeded with 40,000 cells/well the day before). Each well was infected with ~ 20,000 PbmCherry_hsp70_ sporozoites for 6 h. The cells were detached with accutase (Innovative Cell Technology), pooled and the equivalent of 10 wells were seeded in 25 cm^2^ cell culture flask. 1/6th of the cells was washed once with PBS and pelleted by centrifugation (2 min 100 g). The pellet was loosened by flicking and the cells were resuspended with 250 μl of RNAlater and stored at 4 °C till all time points were harvested. It has been reported that RFP in contrast to GFP is preserved in RNAlater treated cells [31].

Media of the cultured cells were changed at 24 hpi and 48 hpi. At the respective time points the cells were detached from the surface with accutase, washed once with PBS and then resuspended in 250 μl RNAlater and stored at 4 °C. The use of RNAlater shortened the parasite’s time in an unnatural state not being in the incubator in adequate environment and medium down to 10 min compared to 1–2 h in case of sorting fresh cells.

To generate transgenic parasites expressing *gfp* under control of the promoter of PBANKA_1003900 (PBANKA_1003900^GFP^), an episomal PbGFPcon vector was used [32]. First, the PBANKA_1003900 promoter (1.7 kb) was amplified using primers GCTCTACCAATTTTGTGTCAC and GGATCCTTAAAAATTAATTTTGTATAAAATCG and cloned into a pCR2.1-TOPO vector (Invitrogen) and sequenced. Then the *P. berghei elongation factor*-*1α* promoter of PbGFPcon was exchanged for the PBANKA_1003900 promoter (*Eco*RV from pCR2.1-TOPO vector/*Bam*HI) and the *gfp* gene was re-introduced in the correct orientation as a *Bam*HI fragment. Finally the construct (Additional file 2: Fig. S1) was used to transfect the reference wild type *P. berghei* ANKA parasite line (cl15cy1 (ANKAwt)) [19] to generate line 300 (PBANKA_1003900^GFP^). Transfection with the episomal construct and positive selection of transfected parasites with pyrimethamine was performed as described previously [19].

For Microscopy work: confluent HeLa cells in 96-well plate (40,000 cells seeded the day before) were infected with ~ 20,000 PBANKA_1003900^GFP^ sporozoites. Cells were washed and detached 2 h post-infection using accutase and seeded on glass covers in 24 wells. At the indicated time points cells were fixed with 4% PFA in PBS for 10 min, washed with PBS and kept at 4 °C. Nuclei were stained with 1 μM Hoechst 33342 for 20 min, embedded with Dako-mounting medium. Fluorescent microscopy pictures were taken on a Leica DM5500B. Signals were photographed using same exposure settings.

For live cell imaging, infected cells were seeded onto glass bottom dishes (35-20-1.5-N, Cellvis, Mountain View). Live cell microscopy was performed with a Leica DMI6000B epifluorescence microscope equipped with a SOLA-SE-II light source starting at 30hpi.

### FACS sorting

Cells kept in RNAlater were FACS sorted on a BD FACSARIA III, FACSflow was used as sheath fluid. A 561 nm laser was used in combination of 610/20 nm filter detect the infected cells (see Additional file 2: Fig. S2). To obtain maximal purity the sorting was performed using the 4-way-purity mode with 100 microns nozzle. The sorted cells were collected into RNAlater (500 μl in Eppedorf tube). 100,000 non-infected cells at each time point were sorted as negative controls.

### RNA isolation, library preparation, sequencing

Prior to isolation of RNA by ReliaPrep™ RNA Cell Miniprep System RNAlater was removed from the cells by adding an equal volume of ddH_2_O to the cells. The cells were then centrifuged (200 g, 2 min). The RNA from the pelleted cells was extracted according to the manufacturer’s protocol and kept at − 80 °C. RNA extraction and Illumina m RNA-sequencing were performed in duplicates. Following RNA isolation, total RNA was quantified with a Qubit Fluorometer (Life Technologies). Quality of the extracted RNA was checked by the RNA integrity number (RIN), measured using an Agilent 2100 BioAnalyser (Agilent Technologies). The SMARTer™ Ultra Low RNA kit from Clontech was used for the reverse transcription and cDNA amplification according to manufacturer’s specifications, starting with 10 ng of total RNA as input. The Nextera XT kit (Illumina, San Diego, CA, USA) was used for cDNA libraries preparation using 200 pg of cDNA. Library molarity and quality was assessed with the Qubit and Tapestation using a DNA High sensitivity chip (Agilent Technologies). The cDNA libraries were pooled and loaded at 12.5 pM, multiplexed on 4 lanes of HiSeq Rapid PE v2 Flow cells for generating paired reads of 100 bases on an Illumina HiSeq 2500 sequencer (Illumina, San Diego, CA, USA).

### Data processing

Short reads generated in this study were deposited at the European Nucleotide Archive “(http://www.ebi.ac.uk/ena/) and are accessible through the accession number PRJEB23770 (Secondary study accession number: ERP105548)”. Publicly available data was obtained from SRA (SRP027529, ERS092084 and ERS092085). All reads were quality-checked with FastQC (bioinformatics.babraham.ac.uk/projects/fastqc). For the publicly available data, illumina adaptor sequences and low-quality reads were removed with TrimGalore (version 0.4.1 with the parameter-illumina), http://www.bioinformatics.babraham.ac.uk/projects/trim_galore). For the data generated in this study, Nextera transposase sequences and low quality reads were removed with Trimmomatic (version 0.33 with the parameters ILLUMINACLIP:adapters/NexteraPE-PE.fa:2:30:10 LEADING:3 TRAILING:3 SLIDINGWINDOW:5:30 MINLEN:50 [33]. Low complexity reads were removed with fqtrim (version 0.9.4) [34]. For paired-end reads, if only one end was removed, the remaining read end was treated as single-end read. To remove potential contamination with host RNA, all reads were aligned to the human genome (ensembl82) with Bowtie2 (version, 2.2.5) [35]. Single-end reads and read pairs with none of the ends aligning to the human genome were kept and aligned to the *P. berghei* ANKA reference genome (PlasmoDB Release 33) with Subread (i.e. subjunc, version 1.4.6-p5) [36] allowing up to 10 alignments per read (options: -n 20 -m 5 -B 10 -H –all Junctions, always in single-end mode, i.e., ignoring the reverse read-end of paired-end reads). Count tables were generated with Rcount [37] with an allocation distance of 100 bp for calculating the weights of the reads with multiple alignments and a minimal number of 5 hits. Count tables are available in Additional file 1: Table S2. Multireads were included to avoid underestimation of expression of genes with similar sequences (see for example [38, 39]). To ensure that each multiread contributes only once to the final expression values, alignments of multireads were weighted according to the number of unique read alignments within 100 bp of a multiread alignment site (for details, see [37]).

### Differential expression

Variation in gene expression was analysed with a general linear model in R with the package DESeq 2 (version 1.16.1) [40] according to a design with a single factor comprising all different experimental groups. Specific groups were compared with linear contrasts and *P*-values were adjusted for multiple testing [41], (i.e., false discovery rate). Genes with an adjusted *P* value (FDR) below 0.01 and a minimal logFC of 2 were considered to be differentially expressed. Normalized gene expression data for plotting and clustering was likewise obtained with DESeq 2 (version 1.16.1) [40].

### Gene co-expression network

To identify groups of genes with similar expression patterns across the life cycle of *P. berghei*, a gene co-expression network (GCN) was constructed. Therefore an adjacency matrix with pairwise Pearson correlation coefficients was calculated, Fisher’s z-transformation was applied and each pairwise correlation coefficient for being significantly bigger than zero was tested (as described in [42]). *P*-values were adjusted for multiple testing [41] and correlation coefficients with an adjusted *P*-value below 0.001 were identified as significant. The significant pairwise correlation coefficients were then used to construct the GCN. To resolve the community structure of the GCN, a modularity optimization algorithm [43] implemented by the function “cluster_louvain” in the R package “igraph” (version 1.0.1) was used [44]. Communities with less than 11 genes were collapsed into a single “mixed” community (70 communities with a total of 197 genes). The network was visualized with Cytoscape (version 3.5.1, “prefuse force directed layout”) [45]. The GeneIDs per community are listed in Additional file 1: Table S4. It should be considered that the GCN analysis was partly based on already published data and for that reason could not be corrected for possible batch effects. The focus of the current study was the liver stage including the sporozoite stage and detached cells as a final point of liver stage development.

### Gene ontology enrichment

To functionally characterize the network communities or genes found to be differentially expressed, enrichment of gene ontology (GO) terms was tested with topGO (version 3.4.1) [46] in conjunction with the GO annotation available from PlasmoDB [22]. Analysis was based on gene counts (genes in the set of interest compared to all annotated genes) using the “weight” algorithm with Fisher’s exact test (both implemented in topGO). A term was identified as significant if the *P*-value was below 0.05.

### Enrichment of selected gene groups

To test for enrichment of a specific group of genes (e.g., “merozoite invasion genes” from [47]) within a gene set of interest compared to all genes annotated with any of the tested groups, we used Fisher’s exact test (two-by-two contingency table). *P*-values were adjusted for multiple testing [41] and groups with an adjusted *P*-value (FDR) below 0.05 were identified as significant.

## Results and discussion

### High fidelity exo-erythrocytic stage RNA-seq data and sample selection criteria

HeLa cells were infected with *P. berghei* sporozoites that express mCherry under the control of a constitutive Hsp70 promoter, allowing the detection of fluorescent parasites in all developmental stages [7]. Exo-erythrocytic form (EEF) parasites were isolated at different time-points of infection by FACS sorting (6 h, 24 h, 48 h, 54 h and 60 h). At 69 h detached cells and merosomes (DCs/merosomes) were collected from the culture medium supernatant. To preserve RNA integrity during FACS sorting of infected cells, cells were treated with RNAlater [31, 48]. In addition, sporozoite samples were generated by processing infected salivary glands of mosquitoes at day 20 post feeding. For each sample, independent biological duplicates were collected. Following the isolation of total RNAs from infected HeLa cells and from infected salivary glands, libraries were sequenced with an Illumina HiSeq 2500 resulting in 34 to 61 million paired-end reads per sample (Additional file 1: Table S1). After removal of low quality sequences, sequencing adapters and sequences arising from host RNA, reads were aligned to the *P. berghei* ANKA reference genome, resulting in around 0.23 to 21.4 million weighted alignments (hits) within genic regions (Additional file 1: Table S1, raw counts are provided in Table S2). Samples collected 6 h after infection were excluded from further analysis as only low amounts of hits were recovered (22,154 and 35,312 hits) in both biological replicates. The reason is most likely that, at this time-point of infection, parasite transcripts represent only a small fraction compared to host cell transcripts.

4475 transcribed genes were identified (≥ 80 normalized read counts, corresponding on average to 20 RPKMs in at least one developmental stage of the EEF). In a previously reported transcriptome analysis of *P. yoelii* EEF stages using microarrays, about 2000 genes were detected [28]. This exemplifies the higher sensitivity of the Next Generation Sequencing (NGS) compared to array technology [39].

**Fig. 1:**
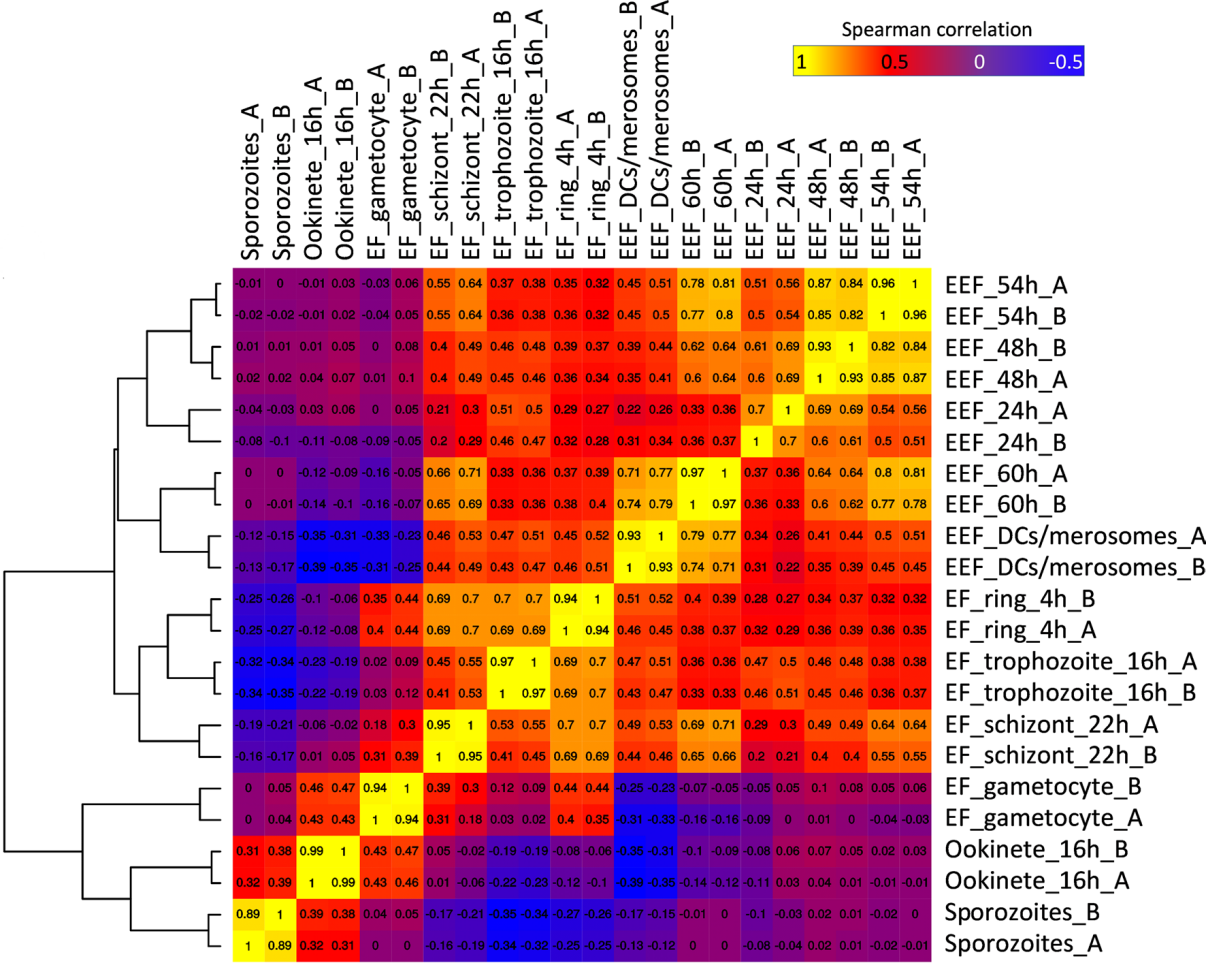
Clustering of samples of different life cycle stages based on genes with the highest overall high variance (90th percentile, Spearman correlation and hierarchical clustering). Stages are sporozoites, exo-erythrocytic (EEF) stages (DCs/merosomes are detached cells and merosomes), erythrocytic (EF) stages (rings, trophozoites, schizonts, gametocytes) and ookinetes. _A, _B: Biological replicates. Heatmap was generated using normalized and log2(x + 1)-transformed gene expression values [40]. Heatmap drawn with the R-package gplots [116]

A hierarchical clustering of the different EEF samples was performed together with RNA-seq data of EF (rings, trophozoites and schizonts harvested 4, 16 and 22 h after infection of RBC), as well as with RNA-seq data of gametocytes and ookinetes [49]. The replicates exhibited a high Spearman’s correlation to each other and the different stages grouped well according to the host environment (exo-erythrocytic, erythrocytic) (Fig. 1).

The analysis revealed that the transcripts profiles of the extracellular ookinetes and sporozoites cluster together, which might be due to the fact, that both are motile stages that traverse mosquito host cells. Ookinetes and sporozoites are, however, markedly different from the profiles of EEF and asexual EF stages. Notably, the expression profile of gametocytes is different from both EEF and asexual EF stages but shows similarities to the ookinetes. This is not surprising as in the *Plasmodium* life cycle gametocytes differentiate into ookinetes (via gamete and zygote stages). In female gametocytes various mRNAs are already produced but translationally repressed and only used during development of the zygote and ookinete [50–52]. Among the EEF stages, the highest similarities of gene expression profiles were observed as expected for adjacent stages/timepoints (e.g., 48 h and 54 h). However, the early stages/timepoints (24 h, 48 h and 54 h) were fairly distinct from the later time-points (60 h and detached cells). The asexual EF stages showed a high degree of similarity among them. In fact, the gene expression profile of detached cells, containing the EEF merozoites, was rather distinct from the profile of late stage EF schizonts, containing EF merozoites, although both need to be prepared to invade RBC. It is noteworthy that proteomics data of *P. yoelii*, revealed that 90% of the proteins of late EEF were also detected in the early EF [28].

To verify the RNA-seq data, the expression pattern of selected genes was compared to previously reported patterns. The expression pattern of the housekeeping proteins (GAPDH, actin 1 and alpha-tubulin 1; Additional file 2: Fig. S3), putative proteases (SERA 1 to 5; Additional file 2: Fig. S4), PVM proteins (EXP1, Exp2, UIS3 and UIS4; Additional file 2: Fig. S5), sporozoite surface proteins (CSP and TRAP; Additional file 2: Fig. S6), fatty acid biosynthesis enzymes (FabB/F, FabI, FabZ and FabG, Additional file 2: Fig. S7) as well as merozoite surface proteins (MSP 1, 4/5, 7, 8, 9, 10; Additional file 2: Fig. S8) were very similar to the already published data confirming the quality of the here presented RNA-seq data. Further information about the selected genes presented in Additional file 2: Figs. S3 to S8 is detailed in the supplementary information section.

Importantly, the expression profiles deduced from the RNA-seq analysis provide valuable information for the choice of promoters to drive expression of stage-specific transgenes, such as fluorescent or luminescent reporter proteins. Previously, the promoters of the housekeeping genes heat shock protein (*hsp70*) and eukaryotic elongation factor 1α (e*ef1α*) have been used to drive expression of fluorescent reporters [7, 32, 53]. According to the here presented RNA-seq analysis, the *hsp70* promoter is a better choice for driving constitutive expression of reporters as *hsp70* mRNA exhibits a more uniform expression profile compared to e*ef1α* mRNA (Additional file 2: Fig. S9).

Next, a more detailed computational analysis of the exo-erythrocytic stage transcriptome and a comparison with other developmental stages was performed.

### Gene co-expression network

**Fig. 2:**
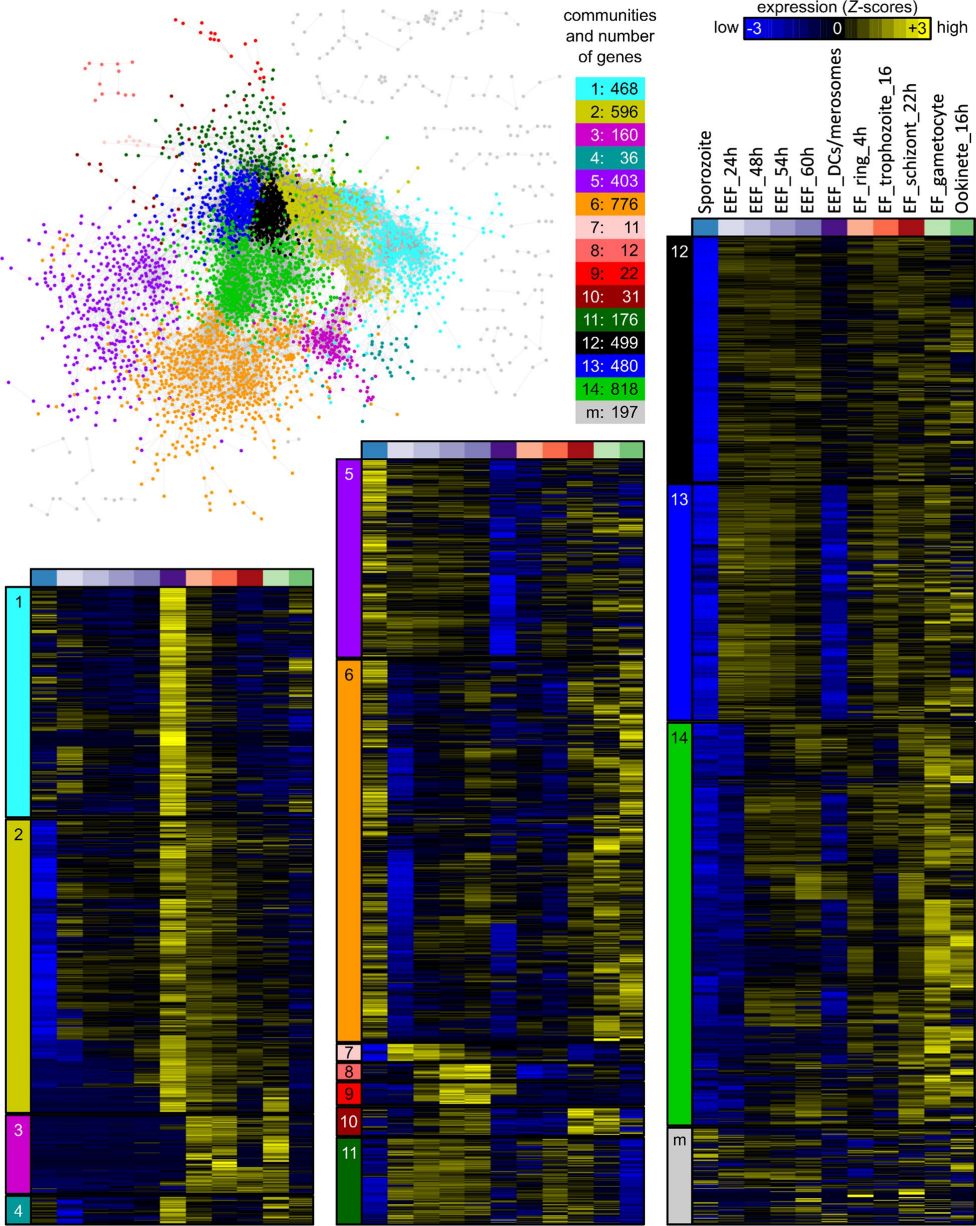
Gene co-expression network based on RNA-seq data of samples of different life cycle stages. Stages are sporozoites, exo-erythrocytic (EEF) stages (DCs/merosomes correspond to detached cells and merosomes), erythrocytic (EF) stages (rings, trophozoites, schizonts, gametocytes) and ookinetes. Each node represents a gene and each edge depicts a significant pairwise correlation. The network was visualized with Cytoscape [45] using the “prefuse force directed layout”. Nodes/genes are colored according to their membership in 14 communities and a ‘mixed’ (M) community (pool of communities with less than 11 genes per community), identified with a modularity optimization algorithm [43]. For each community, a heatmap summarizes the expression patterns of all genes within the community. Expression values in the heatmaps correspond to gene-wise *Z*-scored of normalized and log2(x + 1)-transformed count data averaged across the replicates

To further explore the complexity of the parasite transcriptome, in particular the gene expression similarities among the different developmental stages, a gene co-expression network (GCN) was computed [42] and genes with similar expression patterns (“communities”) [43] were extracted and visualized (Fig. 2). This analysis allowed to gain insight into the sets of similarly expressed genes in the different EEF and EF stages and into characteristic expression patterns within the entire transcriptome. These analyses were used to find functionally related genes based on similar expression patterns.

**Fig. 3:**
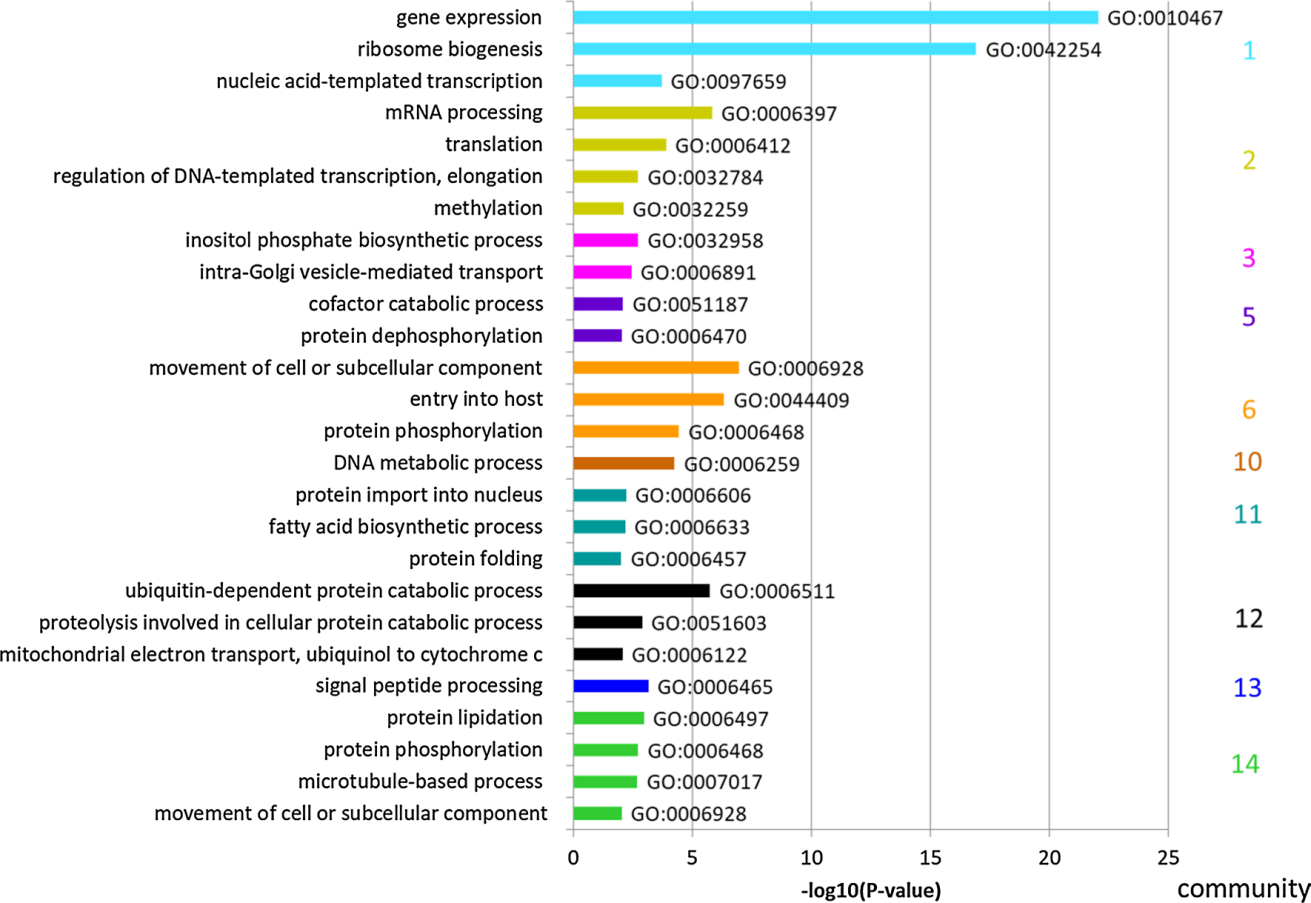
GO terms in GCN communities (expressed as − log10(P-value)). The colors refer to the different communities in Fig. 1b. Only GO-terms with P-values < 0.01 were included. Communities containing less than 25 genes were ignored because of potential false significance (following recommendations in GeneSetEnrichmentAnalysis from the Broad Institute)

The GCN analysis revealed 14 different communities comprised of 11 to 818 genes and a “mixed” community with 197 genes (a pool of communities with 10 or less genes per community) (Fig. 2, Additional file 1: Table S4). Of a total of 5104 genes, 4675 genes are represented in the GCN. 429 genes did not meet the GCN criteria, as the expression pattern of each of them did not significantly correlate to the expression pattern of another gene throughout all stages. Interestingly, the GCN analysis indicated marked differences between EEF and EF. Unexpectedly, even the transcriptome of EEF-derived and EF-derived merozoites (i.e., detached cells and late blood schizonts) were found to differ substantially. The 486 transcribed genes of community 1 were strongly expressed in DCs/merosomes, which contain EEF-derived merozoites and extended into the ring stage (initial phase of EF development). The 596 transcribed genes of community 2 were as well enriched in DCs/merosomes, but expression of these genes persisted longer during the EF (into trophozoite and partly into schizont stage) whereas their expression was strongly reduced in the sporozoites. To functionally characterize the communities defined in the GCN, a gene ontology (GO) term enrichment analysis from the domain “Biological Process (BP)” was performed (Fig. 3, Additional file 1: Table S3).

The first two communities in Fig. 2 contain genes that are expressed specifically in DCs/merosomes and EF stages. The majority of the corresponding gene products were predicted to be involved in gene expression, ribosome biogenesis and transcription (Community 1), or in mRNA processing and translation (Community 2) (Fig. 3). All these functions are involved in DNA/RNA biology, particularly in gene expression and regulation. This is not surprising as invasive forms like the merozoites in DCs/merosomes prepare for the next growth phase after invasion and most likely need stored transcripts for a rapid protein synthesis after invasion of erythrocytes, comparable to the storage of repressed transcripts in mature gametocytes and sporozoites [50–52, 54–58]. On the other hand, the 160 genes of community 3 were highly specific to the EF stages (Fig. 3). Conspicuously, this community has almost no GO-term annotations for biological processes (only 3 out of 160 genes were annotated). However, this community was highly enriched for small nucleolar RNAs (snoRNAs), PIR pseudogenes (*Plasmodium* interspersed repeat pseudo genes) and genes of the three large multigene families in rodent parasites, coding for PIR proteins, fam-a proteins and fam-b proteins. SnoRNAs are important components of ribosome biogenesis that consist in non-coding RNAs with a diversity of function like pseudo-uridylation and 2′-O-methylation of RNAs or synthesis of telomeric DNA [59]. PIR, fam-a and fam-b proteins are exported by EF stages into the cytoplasm of the host erythrocyte. Recently it has been shown that a subset of PIR, fam-a and fam-b proteins are also expressed in EEF stages [60], however the function of most of these proteins remains unknown.

Community 6, consisting of 776 genes, was enriched for genes expressed in sporozoites, but also frequently expressed at elevated levels in gametocytes, ookinetes and schizonts. Not surprisingly, genes preferentially expressed in community 6 were involved in host cell entry, host cell exit and parasite motility (Fig. 3). In addition, genes that are involved in transport of subcellular components and DNA repair were present. Genes whose expression was found to be more specific to sporozoites, with persisting expression during the early EEF stages, but almost complete absence in DCs/merosomes (community 5), were involved in DNA synthesis and metabolic processes, consistent with the high multiplication observed following hepatocyte invasion by the sporozoites (Fig. 3).

Communities 7, 8 and 9 contain 45 genes in total, the expression of which were mostly specific to the developing EEF stages. According to guidelines of the Broad Institute on GeneSetEnrichmentAnalysis, small size communities should not be interpreted.

The expression of the 31 genes of community 10 was enriched in EF schizonts and in gametocytes, but these genes were also found well expressed in late EEF stages. Although the GO term ‘DNA metabolic process’ is listed for this community, it should be assessed with caution due to the reason mentioned above.

The 176 genes of community 11 were expressed during the developing EEF and EF stages, with a slight bias towards the developing EEF stages (85% of all genes in the community were on average expressed at a higher level in the EEF stages). In this community, 3 out of 9 genes coding for enzymes of fatty acid biosynthesis have been identified as hits. This is in agreement with the high fatty acid usage of EEF stages to generate various parasite membranes [61]. Apart from genes involved in fatty acid biosynthesis, it is very likely that genes identified in this community are involved in schizogony and merozoite development in both EEF and EF stages.

The remaining communities were mostly defined by genes with almost complete absence of expression in sporozoites (community 12; 499 genes), in sporozoites and DCs/merosomes (community 13; 480 genes) or in sporozoites, 24 h EEF stage and partly DCs/merosomes (community 14; 818 genes). However, whereas genes of community 12 and 13 were generally expressed throughout the EEF and the EF stages, genes of community 14 were more specific to gametocytes and ookinetes (Fig. 2). Sporozoites are not growing or proliferating and therefore it can be expected that in sporozoites, expression of genes involved in several metabolic processes, protein lipidation, phosphorylation and signal peptide processing is less pronounced than in other stages.

Altogether, the GCN analysis allowed to identify 14 clearly defined communities and a pool of small communities (mix) with totally 4675 genes attributed (see Additional file 1: Table S4 for GeneIDs of the members of the communities and for genes excluded during the GCN analysis).

The generated GCN provides a first comprehensive overview of gene regulation in a *Plasmodium* parasite throughout EEF and EF development including several life cycle stages in the mosquito vector (ookinetes, sporozoites). The identification of clear communities of genes with comparable expression profiles may help identifying common signatures in the untranslated promotor regions that may be involved in regulation of gene expression.

### Differences in gene expression between developing exo-erythrocytic and erythrocytic parasites

To better elucidate the differences between EEF and EF stage parasites, differential expression analyses were conducted. A limitation of this approach was that blood stage gene transcription was based on already published data, which cannot be corrected for batch effects or contaminations with sexual stage parasites. Known confounders are the contamination of the 4 h EF ring stage with about 3% mature gametocytes and the 22 h EF schizont cultures with about 10% immature gametocytes [49]. In addition, the transition between ring forms and young trophozoites is not exactly defined by morphological or molecular means. Apart from these reasonable limitations, a comparison of the highly enriched and homogeneous stages during blood and liver stages revealed very interesting differences.

**Fig. 4:**
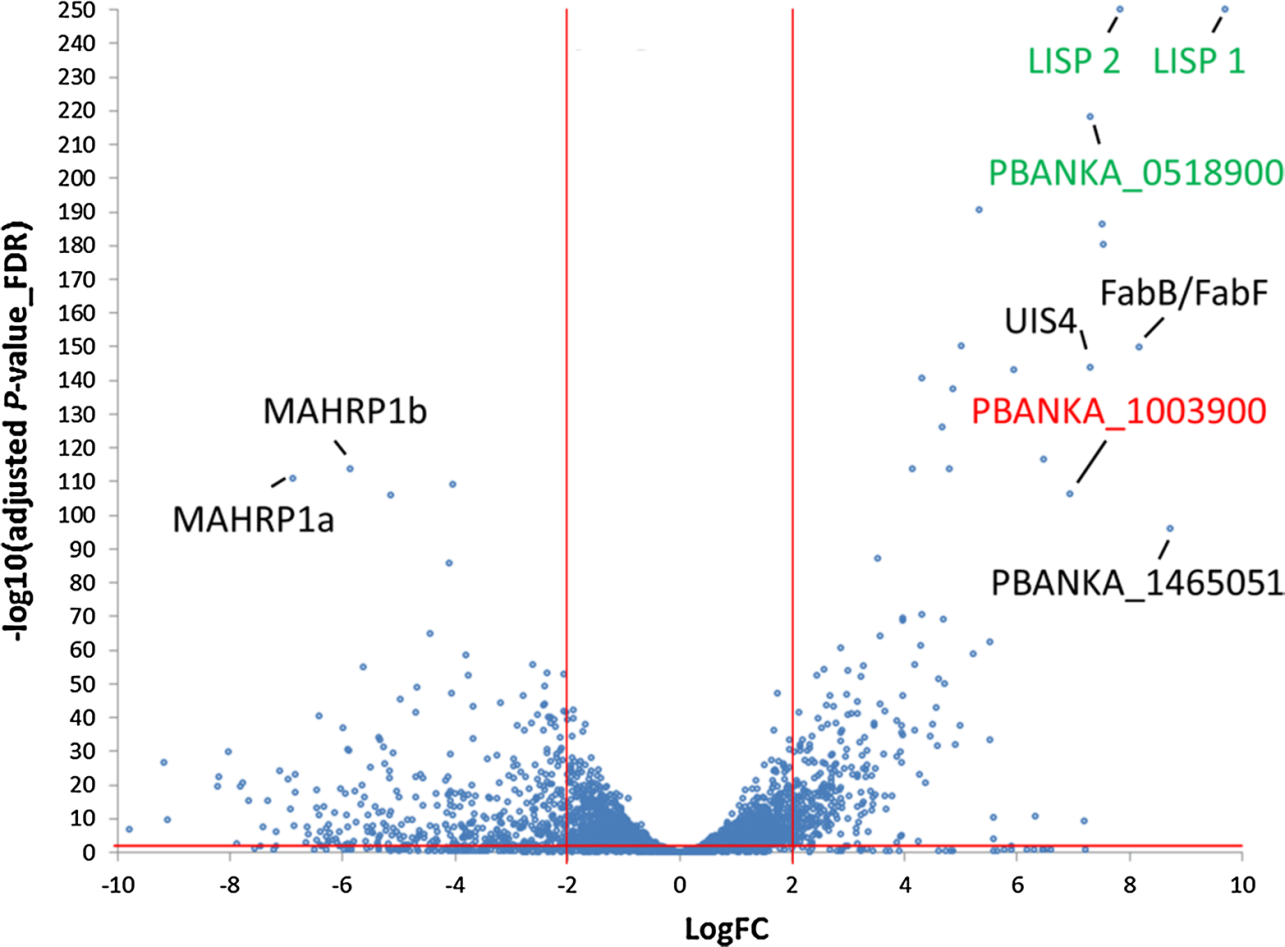
Volcano plot of differently expressed genes of developing exo-erythrocytic (EEF) stages (EEF_24 h–60 h) compared to developing erythrocytic stages (EF_ring, EF trophozoites). In this analysis the DCs/merosomes and schizonts stages are not included. The graph shows LogFC values relative to FDR (− Log10(adjusted P-value). Positive LogFC values represent preferentially EEF stage expressed genes. Negative LogFC values represent preferentially EF stage genes. Genes in green and red show highest expression in liver stages compared to all other stages (including sporozoites, gametocytes and ookinetes)

In order to compare EF and EEF parasites, averaged gene expression of developing EEF stages (24 h, 48 h, 54 h and 60 h) and of developing EF stages (ring 4 h, trophozoite 16 h) was compared, despite that such an averaging bears a potential bias towards earlier or late stages. In this comparison, 299 genes were significantly upregulated in the EEF stages (LogFC ≥ 2, adjP ≤ 0.01) and 392 genes were significantly upregulated in the EF stages (Fig. 4; Additional file 1: Table S5). GO-term enrichment (summarized in Table 2) revealed that genes preferentially expressed in the EEF stages were enriched for the fatty acid biosynthesis pathway (e.g. *fabB*/*fabF* in Fig. 4), entry into the host cell, leading strand elongation, tetrapyrrol biosynthesis and DNA replication. Considering that a single *P. berghei* EEF parasite generates more than 10,000 merozoites and an individual EF stage parasite only 12–18, an enrichment of expression in genes associated with fatty acid biosynthesis and DNA replication is expected and has already been confirmed in other studies [62, 63] and reviewed in [64]. In contrast, genes preferentially expressed in the EF stages were enriched for the GO terms for cell motility and intracellular, organellar transport (summarized as “movement of cell or subcellular components”), protein export into host cell cytoplasm, exit from host cell and pathogenesis. Enrichment of gene expression related to translocation of proteins (e.g. membrane associated histidine-rich protein 1: *mahrp1a* and *b* in Fig. 4) across the PVM is also required for parasite remodeling of the host RBC [65], including proteins transported to the surface of the RBC involved in RBC sequestration [65–67]. Many *P. berghei* proteins are known to be transported into the host red blood cell [60, 67] whereas only a few proteins have been identified to be exported into the host hepatocyte, for example, CSP [68, 69] and LISP2 [70].

**Table 2:** Gene ontology (GO) term annotation of genes preferentially expressed in (i) EEF stages and (ii) asexual EF stages

Biological process ID	Process description	Annotated	Observed	P value
***(i) Preferentially expressed in exo-erythrocytic stage (24 h, 48 h, 54 h and 60 h)***				
GO:0006633	Fatty acid biosynthetic process	9	6	4e−06
GO:0044409	Entry into host	33	7	0.0036
GO:0006272	Leading strand elongation	2	2	0.0040
GO:0033014	Tetrapyrrole biosynthetic process	9	4	0.0042
GO:0006260	DNA replication	39	10	0.0110
GO:0055114	Oxidation–reduction process	83	12	0.0215
GO:0006270	DNA replication initiation	4	2	0.0219
GO:0006334	Nucleosome assembly	4	2	0.0219
***(ii) Preferentially expressed in erythrocytic stage (ring 4 h, trophozoite 16 h)***				
GO:0006928	Movement of cell or subcellular component	34	6	0.00036
GO:0044053	Translocation of peptides or proteins into host cell cytoplasm	3	2	0.00256
GO:0040011	Locomotion	22	5	0.00644
GO:0035891	Exit from host cell	8	2	0.02169
GO:0009405	Pathogenesis	8	2	0.02169
GO:0030833	Regulation of actin filament polymerization	1	1	0.02978
GO:0006166	Purine ribonucleoside salvage	1	1	0.02978
GO:0019510	*S*-Adenosylhomocysteine catabolic process	1	1	0.02978
GO:0010323	Negative regulation of isopentenyl diphosphate biosynthetic process, methylerythritol 4-phosphate pathway	1	1	0.02978
GO:0020035	Cytoadherence to microvasculature, mediated by symbiont protein	1	1	0.02978
GO:0007050	Cell cycle arrest	1	1	0.02978

The number of annotated genes (number genes per GO term present in the entire RNA-seq study) and observed genes (number of genes per GO term preferentially expressed in…) per Biological Process are listed. Only GO terms with P-values < 0.05 are shown

### Exo-erythrocytic and erythrocytic merozoites: same but different

**Fig. 5:**
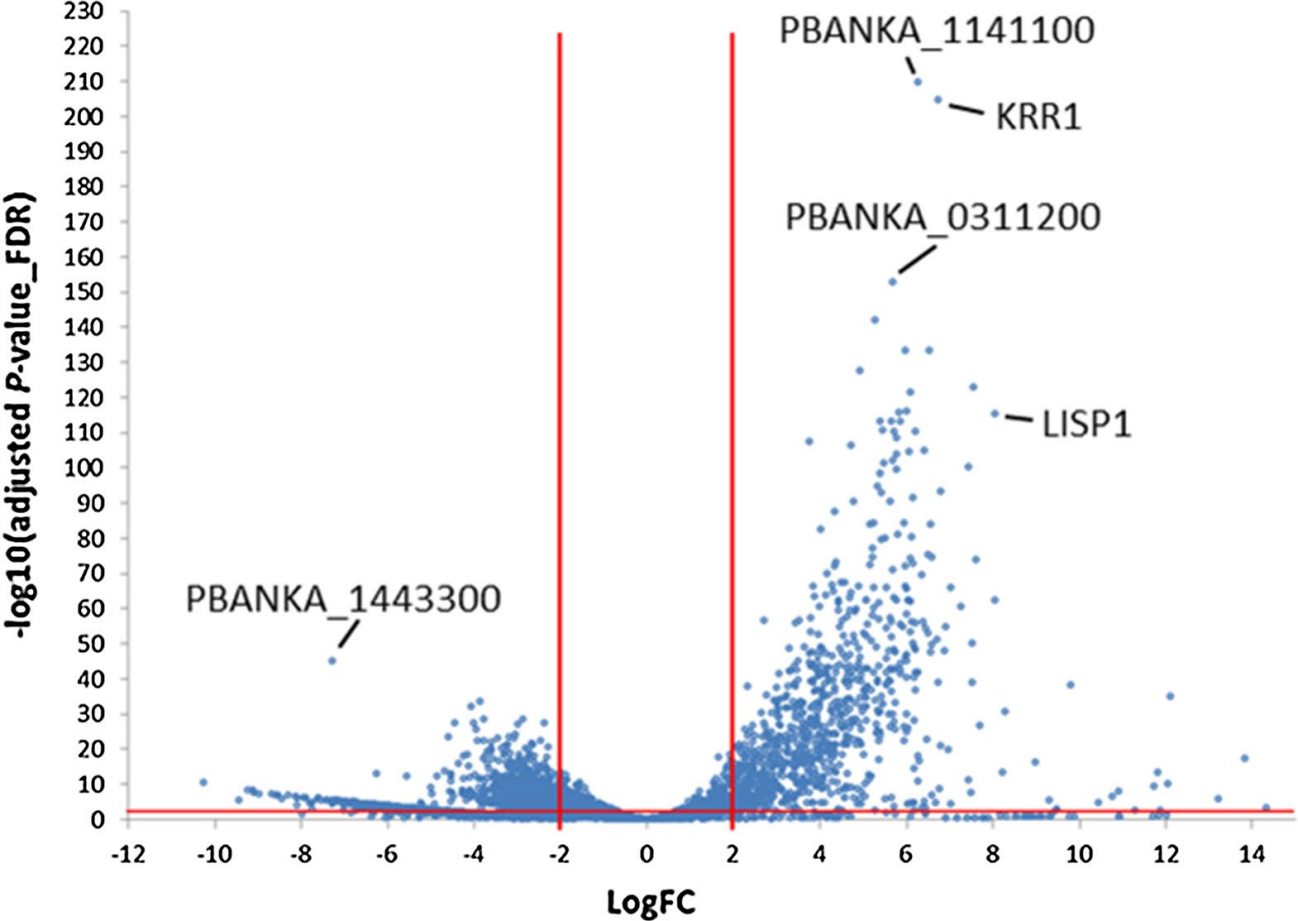
Volcano plot of differently expressed genes of detached cells and merosomes compared to EF schizonts (sample 22 h). Positive LogFC values represent preferentially DCs/merosomes expressed genes. Negative LogFC values represent preferentially EF schizont expressed genes. The graph shows LogFC values relative to FDR (− Log10(adjusted P-value)

In contrast to the limited differences in gene expression observed during parasite development in hepatocytes and RBC, the differences between first generation merozoites generated at the end of the liver stage and subsequent generation merozoites generated during the repeated rounds of blood stage development, were much more pronounced (i.e. see communities 1, 2, 4, 5, 10, 13 in Fig. 2) even though the 22 h EF schizont sample was not entirely pure but also contained some immature schizonts and immature gametocytes as stated in the corresponding publication [49]. Still, 880 and 1275 genes were preferentially expressed in DC s/merosomes and in 22 h schizonts, respectively (Fig. 5 and Additional file 1: Table S6). GO-term enrichment analysis (Table 3) indicated clear differences between these sets. Genes preferentially expressed in first generation merozoites (DCs/merosomes) were found enriched for the GO-terms: gene expression, ribosome biogenesis, amide biosynthetic process, RNA biosynthetic process and mRNA splicing. In contrast, genes preferentially expressed in subsequent generation merozoites (22 h EF schizonts) were enriched for the GO-terms: small GTPase-mediated signal transduction, DNA replication and recombination, protein localization and modification, and signal peptide processing. At first glance, it is rather surprising that merozoites derived from EEF and EF stage differ so markedly. However, it might reflect the fact that the mechanism of egress from their respective host cells differ markedly. EF stage-derived merozoites almost simultaneously rupture the PVM and the plasma membrane of the RBC [17]. On the other hand, EEF stage-derived merozoites are initially freed from the PVM but can then stay for several hours in the host cell until they are extruded inside merosomes into a blood vessel [7, 14] and are eventually released from merosomes in the fine capillaries of the lungs [16]. Also, DCs/merosomes do not form entirely synchronously. In vitro generation of detached cells starts as early as 54 h, peaks at 65 h but continues until 69 h and even later. Since for the current study DCs/merosomes were collected at 69 h to increase the yield, some merozoites might be at different developmental and activation stages and thus might express different sets of genes. However, the vast majority of 22 h EF schizonts and 69 h DCs/merosomes contain fully matured and infectious merozoites and thus are certainly the best defined stages to compare between blood and liver stages. The next section focuses in more detail on genes involved in the egress of EEF stage-derived merozoites in comparison to EF stage-derived merozoites.

**Table 3:** Gene ontology (GO) term annotation of genes preferentially expressed in (i) EEF merozoites and (ii) EF merozoites

Biological process ID	Process description	Annotated	Observed	P value
***(i) Preferentially expressed in exo-erythrocytic stage merozoites (DCs/merosomes)***				
GO:0010467	Gene expression	462	181	3.0e−22
GO:0042254	Ribosome biogenesis	63	47	4.6e−20
GO:0043604	Amide biosynthetic process	219	98	1.1e−17
GO:0032774	RNA biosynthetic process	103	35	0.00017
GO:0017183	Peptidyl-diphthamide biosynthetic process from peptidyl-histidine	5	4	0.00700
GO:0000398	mRNA splicing, via spliceosome	43	15	0.01718
GO:0008295	Spermidine biosynthetic process	2	2	0.04105
GO:0008612	Peptidyl-lysine modification to peptidyl-hypusine	2	2	0.04105
***(ii) Preferentially expressed in erythrocytic stage merozoites (22 h schizonts)***				
GO:0007264	Small gtpase mediated signal transduction	26	14	0.0014
GO:0006260	DNA replication	39	20	0.0049
GO:0045184	Establishment of protein localization	93	34	0.0060
GO:0007017	Microtubule-based process	35	15	0.0075
GO:0000041	Transition metal ion transport	7	6	0.0151
GO:0006465	Signal peptide processing	5	4	0.0153
GO:0006848	Pyruvate transport	3	3	0.0154
GO:0006497	Protein lipidation	21	10	0.0196
GO:0006511	Ubiquitin-dependent protein catabolic process	38	16	0.0248
GO:0043248	Proteasome assembly	8	5	0.0267
GO:0006310	DNA recombination	9	6	0.0366
GO:0006468	Protein phosphorylation	82	28	0.0437

The number of annotated genes (number genes per GO term present in the entire RNA-seq study) and observed genes (number of genes per GO term preferentially expressed in…) per Biological Process are listed. Only GO terms with P-values < 0.05 are shown

### Comparison of mRNA expression patterns at the end of exo-erythrocytic and erythrocytic stage development

Given that EF merozoites egress within minutes upon PVM rupture, whereas EEF merozoites remain in the hepatocyte cytoplasm for up to several hours, genes were searched that might be (i) necessary for the rapid egress specifically from RBC, (ii) required for survival in the hepatocyte cytoplasm and (iii) specific for both developmental stages (i.e. necessary for PVM rupture in RBC and hepatocytes). Interestingly, 74 genes were upregulated in 22 h EF schizonts in comparison to EEF samples (LogFC ≥ 2, adjP ≤ 0.01, Additional file 1: Table S7), but only four of these were upregulated when compared to all other stages (Additional file 1: Table S8). These four genes code for the following proteins: (i) the high mobility group protein B1 (PBANKA_0601900) of which the orthologue in *P. falciparum* has been shown to potently activate pro-inflammatory cytokines, suggesting a role in triggering host inflammatory immune responses [71]; (ii) a protein of the PIR multigene family (PBANKA_0500781); the function of PIRs is not known but these proteins are believed to be involved in antigenic variation and evasion from host immune responses [72]; (iii) a conserved *Plasmodium* protein of unknown function (PBANKA_0915200); (iv) MSP9 (Merozoite surface protein 9, PBANKA_1443300). The orthologue of MSP9 in *P. falciparum* has been shown to bind to erythrocyte band 3 protein and to form a complex with MSP1 [73, 74]. *Plasmodium falciparum* merozoites are able to infect RBC via two different invasion mechanisms: one is sialic acid-dependent, involving MSP9 and the other one is MSP9 and sialic acid-independent. Interestingly *msp9* is barely transcribed in DCs/merosomes and also hardly in other developmental EEF stages, which might be indicative that EEF stage-derived merozoites do not invade in the sialic acid-dependent manner. Attempts to knock out *P. berghei* MSP9 were not successful [75]. In *P. yoelii*, MSP9 has been found in the erythrocyte cytoplasm [76] and might, in addition to invasion, also be involved in egress of merozoites from RBC.

Thereafter, 293 genes were identified that are specifically upregulated in DCs/merosomes when compared to all other stages (Additional file 1: Table S9). It is conceivable that most of them are involved in the EEF stage-specific egress, in parasite survival in the dying host hepatocyte or in early RBC remodeling. As discussed earlier, it is plausible that transcripts are translationally suppressed in merozoites until the next stage in the life cycle, like has been described for mature gametocytes and sporozoites [51, 77]. Among the highly upregulated transcripts, *sbp1* (skeleton binding protein 1) (PBANKA_1101300), *mahrp1a* and *mahrp1b* (PBANKA_1145800 and PBANKA_1145900) were identified. These gene products are all involved in the trafficking of exported proteins from the parasite to the surface of the RBC [65]. Knock out of MAHRP1a or SBP1 reduced the sequestration of infected cells [65]. It is plausible that EEF stage-derived merozoites express high levels of these mRNAs in order to sufficiently express these proteins immediately after infection of RBC. Plausibly, this may allow the infected RBC to be efficiently sequestered in the periphery to avoid immediate clearance by the spleen. Why expression of *sbp1* and *mahrp1a* and *mahrp1b* appears to be lower in EF stage merozoites than in EEF stage merozoites is unknown. One reason might be, that in course of EF stage infection, the spleen is heavily remodeled [78–80] and allows for passage of infected RBC, making an efficient sequestration and thus a pronounced expression of sequestration ligands on the surface of the infected RBC less necessary [65]. The parasite might thus be less dependent on intense trafficking of sequestration ligands. Another reason might be that *mahrp1a* and *sbp1* are already expressed during ring and trophozoite stage and thus less transcript would be needed to fulfill the same function as in EEF.

### Genes predominantly expressed in exo-erythrocytic stages

**Fig. 6:**
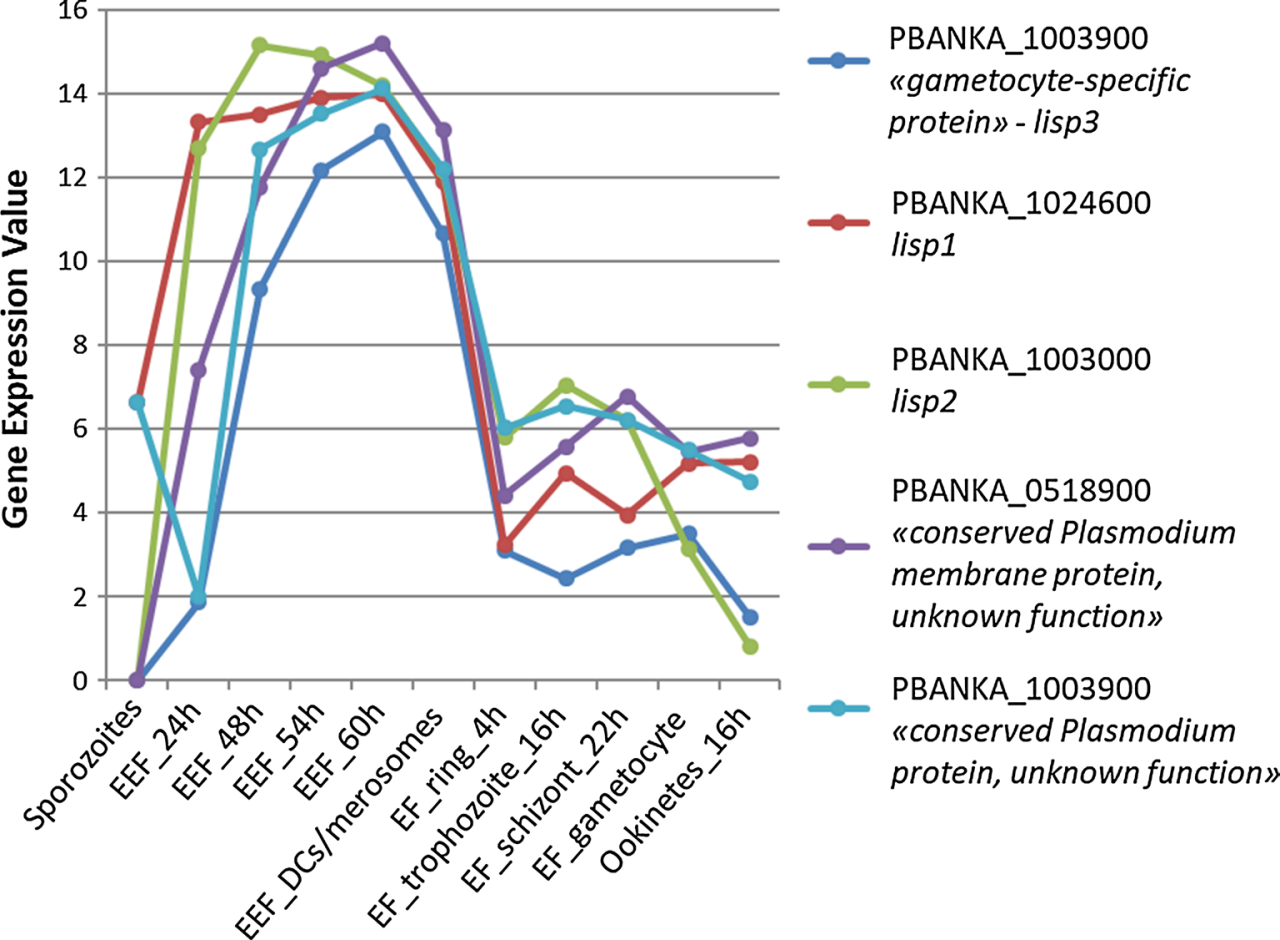
Expression profiles of the top 5 genes predominantly expressed in exo-erythrocytic stages compared to all other stages. Gene expression values corresponding to normalized and log2(x + 1)-transformed read counts. The data were normalized with DESeq 2 (with default parameters) [40]

One of the goals of this study was to identify EEF stage-specific expressed genes. The EEF data were therefore compared with data from all other stages. The comparison revealed 5 highly specifically expressed transcripts for EEF stages with a LogFC > 6 and adjP < 0.01 (Fig. 6). The genes *lisp1* (PBANKA_1024600) *and lisp2* (PBANKA_1003000) have been previously reported to be expressed exclusively during EEF stage development [80–82]. This is clearly reflected by the here presented RNA-seq analysis where substantial *lisp1* and *lisp2* mRNA levels were only found in EEF stages, from 24 hpi to DCs/merosomes. Along with *lisp1* and *lisp2* two conserved *Plasmodium* genes (PBANKA_0518900 and PBANKA_0519500) were identified, one of which is annotated as membrane protein, although there is no transmembrane domain other than the signal peptide (PBANKA_0518900). The fifth gene in this EEF-specific group of genes is PBANKA_1003900. An averaged logFC of PBANKA_1003900 from later stages compared to non-EEF stages was similarly high as for *lisp2* and *lisp1* (Table 4).

**Table 4:** LogFC and FDR (adjP) values of EEF-specific genes

		48 h to DCs/merosomes compared to other stages		
		Mean LogFC	Min adjP	Max adjP
Liver specific protein 2 *(lisp2)*	PBANKA_1003000	9.946	2.26E−228	1.22E−18
Liver specific protein 3 *(lisp3)*	PBANKA_1003900	8.730	9.44E−133	2.17E−10
Conserved *Plasmodium* membrane protein, unknown function	PBANKA_0518900	8.697	3.36E−175	9.55E−17
Liver specific protein 1 *(lisp1)*	PBANKA_1024600	8.475	1.21E−189	1.24E−38
Conserved *Plasmodium* protein, unknown function	PBANKA_0519500	7.035	7.81E−64	2.28E−21

LogFC is indicated as mean of EEF late stages by single comparisons to each other stage. FDR (adjP) values are presented as min and max values of the different comparisons

**Fig. 7:**
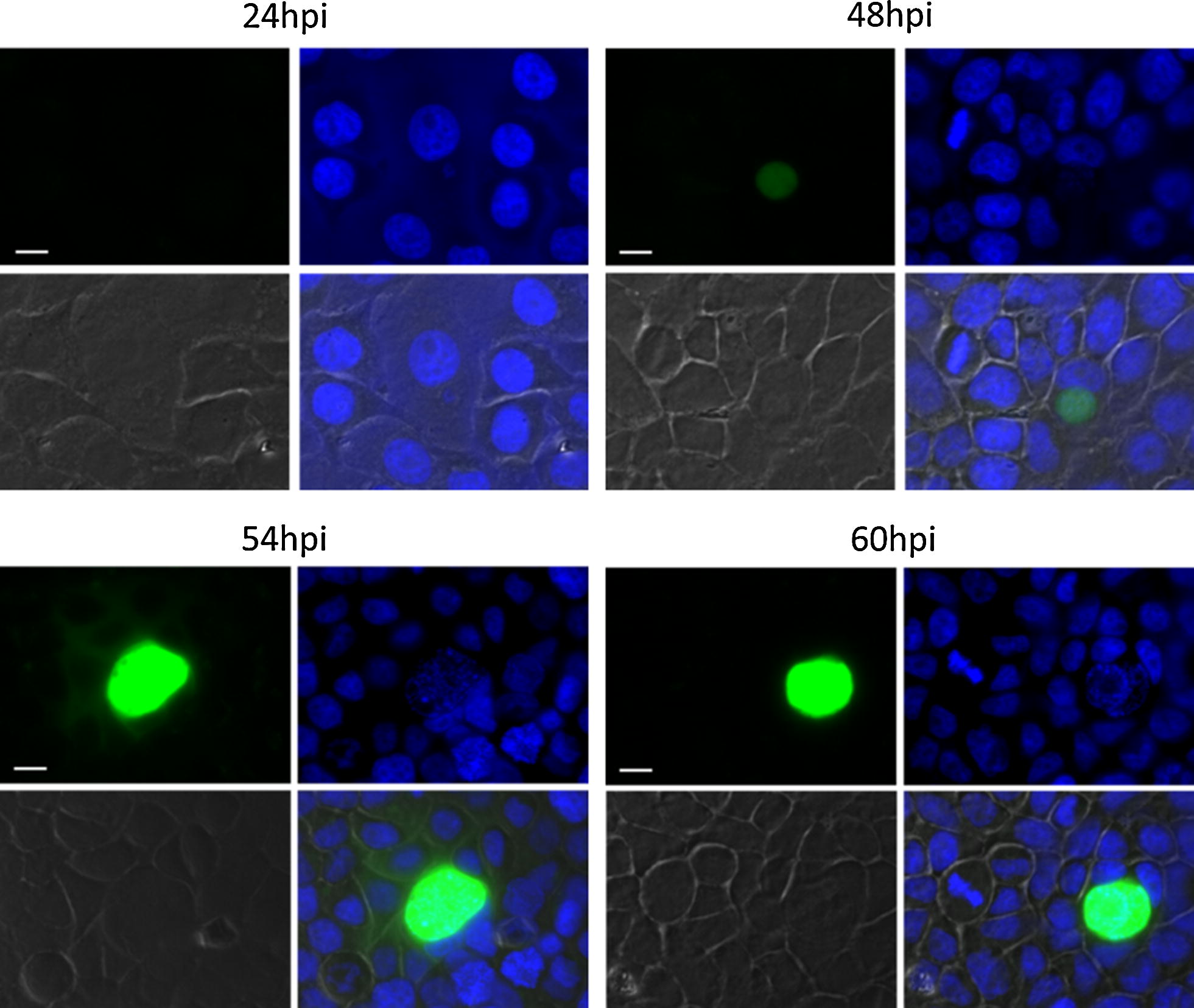
GFP expression during EEF stage development of the transgenic line 300 expressing GFP under control of the promoter region of PBANKA_1003900 (PBANKA_1003900^GFP^). Infected HeLa cells were fixed with 4% PFA/PBS at indicated times post infection with sporozoites. Nuclei were stained with Hoechst. Microscopy-settings (e.g. exposure time) were kept the same for all samples (bar 10 µm)

PBANKA_1003900 is a syntenic ortholog of *P. falciparum* sexual stage-specific protein precursor (Pfs16; PF3D7_0406200) which is expressed early during development of *P. falciparum* gametocytes [83]. *Plasmodium berghei* parasites expressing an mCherry-tagged PBANKA_1003900 provided experimental evidence that this gene is also expressed in gametocytes and was, therefore, annotated as a gametocyte specific protein [84]. However, substantial PBANKA_1003900 transcript levels were only detected in EEF stages. A transgenic parasite line expressing GFP under the control of the promoter of PBANKA_1003900 (PBANKA_1003900^GFP^) was generated and analysed for GFP expression by fluorescence microscopy in the different EEF and EF stages. GFP was not detectable in any of the EF stages, including gametocytes. In contrast, GFP expression was detected by fluorescence microscopy of infected HeLa cells fixed at different time points (Fig. 7; 24 h, 48 h, 54 h, 60 h). At 24 h of EEF stage development no or only very weak GFP-fluorescence was detectable. At 48 h of EEF stage development, the signal was readily visible and at 54 h and 60 h post infection the fluorescent signal was profoundly intense. When performing live cell imaging, the first signals were observed at 30 h post infection (Additional file 3: Movie S1, starting from 30 hpi). From 45 h onwards the protein was substantially expressed confirming the results obtained from fixed cells. These fluorescence patterns (fixed and live) nicely confirmed the RNA-seq data during EEF stage development. Interestingly, analyses of gene-deletion mutants lacking PBANKA_1003900 demonstrated that the gene is not essential at any developmental stage throughout the complete parasite life cycle ([84] and own unpublished data). According to the expression profile deduced from the RNA-seq analysis and also confirmed by the promoter analyses, it is appropriate to revise the annotation as “gametocyte specific protein” and to rename it as “liver specific protein 3 (LISP3)”.

## Conclusion

This study presents a time-course transcriptomic assessment of *P. berghei* liver stage development with emphasis on late stage EEF stages and an extended comparative gene transcription analysis including published transcriptomic data from erythrocytic stages (sexual and asexual), as well as the ookinete stage. This offers a comprehensive overview of gene transcription throughout most of the life cycle and allows a better understanding of gene regulation in different life cycle stages. In particular, the transcriptome of these different life cycle stages provides an invaluable tool for systems biology approaches to model metabolic pathways that are essential in different steps of the *Plasmodium* life cycle. In a first attempt to analyse transcription profiles during the *Plasmodium* life cycle, almost 4700 genes were segemented into 14 distinct communities based on their different expression profile and attributed gene ontology terms to the individual communities. A more detailed analysis of the promoter regions of the genes in the communities with similar expression profiles could help identifying common DNA domains that might support the further understanding of regulation of gene expression in *Plasmodium*. The RNA-seq data provided here are of considerable interest for fundamental research questions with respect to the parasite biology as well as for applied research at the identification of new protein targets for vaccine and drug development.

## Supplementary information

**Additional file 1: Table S1.** RNA-seq data of different life cycle stages of *P. berghei* used in this study. **Table S2.** RNA-seq analysis: Raw sequencing counts per gene in the different life cycle stages. **Table S3.** Gene ontology (GO) term annotation of genes of the individual communities in the GCN. **Table S4.** *P. berghei* geneIDs of genes of the different communities in the GCN. **Table S5.** Genes preferentially expressed in developing EEF stages compared to developing EF stages. **Table S6.** Genes preferentially expressed in detached cells (DCs/merosomes) compared to erythrocytic schizonts. **Table S7.** Genes preferentially expressed in blood schizonts (22 h) compared to EEF stages. **Table S8.** Genes preferentially expressed in blood schizonts (22 h) compared to all other stages. **Table S9.** Genes preferentially expressed in detached cells (DCs/merosomes) compared to all other stages.

**Additional file 2: Figure S1.** Generation and genotyping of parasites expressing *gfp* under control of the promoter of PBANKA_1003900 (PBANKA_1003900^GFP^). **Figure S2.** Fluorescence-activated cell sorting of infected HeLa cells preserved in RNAlater. **Figure S3.** RNA expression profiles of 3 housekeeping genes (*gapdh, actinI, tubulin1*). **Figure S4.** RNA expression profiles of 5 genes encoding serine-repeat antigens, serine-type proteases (SERA1-5). **Figure S5.** RNA expression profiles of 5 genes encoding proteins of the parasitophorous vacuole membrane (Exported protein 1, Exported protein 2, UIS3, UIS4). **Figure S6.** RNA expression profiles of genes encoding 2 sporozoite surface proteins (CSP and TRAP). **Figure S7.** RNA expression profiles of 4 genes encoding enzymes involved in fatty acid biosynthesis (FabB/F, FabI, FabZ, FabG). **Figure S8.** RNA expression profiles of 6 genes encoding merozoite surface proteins (MSP). **Figure S9.** RNA expression profiles of 3 genes whose promoter regions have been used to drive expression of fluorescent/luminescent reporter proteins (HSP70, two genes for EF1α).

**Additional file 3.** Time laps: GFP expression under the control of PBANKA_1003900 promoter during EEF stage development.

## Data Availability

Short reads generated in this study were deposited at the European Nucleotide Archive (http://www.ebi.ac.uk/ena/) and are accessible through the accession number PRJEB23770 (Secondary study accession number: ERP105548).
