# Supplementary material for: Transcriptome analysis of Plasmodium berghei during exo-erythrocytic development
Source: Malar J. 2019 Sep 24;18:330. doi: 10.1186/s12936-019-2968-7 (PMC6760107; doi:10.1186/s12936-019-2968-7)
Supplement: Supplementary file 2 — Additional file 2: Figure S1. Generation and genotyping of parasites expressing gfp under control of the promoter of PBANKA_1003900 (PBANKA_1003900GFP). Figure S2. Fluorescence-activated cell sorting of infected HeLa cells preserved in RNAlater. Figure S3. RNA expression profiles of 3 housekeeping genes (gapdh, actinI, tubulin1). Figure S4. RNA expression profiles of 5 genes encoding serine-repeat antigens, serine-type proteases (SERA1-5). Figure S5. RNA expression profiles of 5 genes encoding proteins of the parasitophorous vacuole membrane (Exported protein 1, Exported protein 2, UIS3, UIS4). Figure S6. RNA expression profiles of genes encoding 2 sporozoite surface proteins (CSP and TRAP). Figure S7. RNA expression profiles of 4 genes encoding enzymes involved in fatty acid biosynthesis (FabB/F, FabI, FabZ, FabG). Figure S8. RNA expression profiles of 6 genes encoding merozoite surface proteins (MSP). Figure S9. RNA expression profiles of 3 genes whose promoter regions have been used to drive expression of fluorescent/luminescent reporter proteins (HSP70, two genes for EF1α). [file 12936_2019_2968_MOESM2_ESM.docx]

**Additional file 2**

**Supplementary Information (Text, Figures, References)**

In the following section individual genes are discussed and RNA-seq expression profiles compared with already published mRNA or protein expression.

i) House-keeping genes (Fig. S3): GAPDH is often used as control in RT-PCR and Western blot analyses to normalize the expression of a gene [1]. GAPDH is a glycolysis enzyme considered to be constitutively expressed in all metabolically active life cycle stages. The RNA-seq profile shows very low levels in sporozoites and reduced levels in detached cells (DC), containing infectious merozoites. Considering that both sporozoites and merozoites are prepared for invasion and are neither growing nor replicating it can be expected that several metabolic processes are less active and glycolysis might be such a process. It has recently been shown that GAPDH resides on the sporozoite surface and interacts with CD68 on Kupffer cells for cell traversal through them [2,3].

The cytoskeleton proteins actin and tubulin are also often used as controls for RT-PCR and Western blot analyses [4,5]. The RNAseq data revealed that *actin I* and *alpha tubulin 1* are constitutively expressed in all life cycle stages tested.

ii) Genes encoding Serine-repeat antigens (Fig. S4)

Previously reported RT-PCR-based analyses of the putative proteases SERA1-5 suggested distinct expression profiles for the different members of this gene family [4]. *sera5* was predominantly expressed in sporozoites, whereas all other *sera* mRNAs were either not expressed or expressed less during this stage but were found upregulated during the liver and blood stages [4,6,7]. Our RNA-seq analysis showed a very similar expression profile as that described for this gene family during liver stage development with high expression of *sera1 to sera4* during EEF and EF_schizont and low expression during sporozoite, ookinete stage and EF_gametocyte.

iii) Genes encoding parasitophorous vacuole membrane (PVM) proteins (Fig. S5)

Exported protein 1 (EXP1), expressed in blood and liver stages [8–10] is widely used as a marker for the PVM in immunofluorescence assays. According to its localization in the PVM in intracellular stages, high level of expression is expected in intracellular liver and blood stages and a lower expression in the extracellular (motile) stages, such as ookinetes and sporozoites. Our RNA-seq shows indeed nearly absence in sporozoites and ookinetes and high expression in developing blood and liver stages.

The two ETRAM proteins, Upregulated in infective sporozoites 3 (UIS3) and 4 (UIS4) are PVM proteins of EEF stage [11,12]. The RNAseq data showed that in particular *uis4* is strongly upregulated in sporozoites but already at 24 hours post infection, the expression level in the liver drops more than 50fold. This decrease in expression of *uis4* is in line with the published data [13]

v) genes encoding sporozoite surface proteins (Fig. S6)

Sporozoite-specific protein expression has been reported for *csp* and *trap* [14,15]. Our analysis clearly confirms the published stage-specific mRNA profile for both genes. Although there is some basal expression in other stages, dramatically higher levels of *csp* and *trap* mRNA levels were detected in the sporozoites.

v) Genes encoding fatty acid biosynthesis enzymes (Fig. S7)

It is well-established that fatty acid biosynthesis is essential for successful completion of liver stage development of rodent malaria parasites (Vaughan et al., 2009; Yu et al., 2008). In *P. yoelii*, the transcription profile of the four genes coding for enzymes related to fatty acid biosynthesis (*fabB/F, fabI, fabZ, fabG*) exhibit a very strong upregulation during liver stage development (Vaughan et al., 2009). Analysis of the same genes in *P. berghei* revealed upregulated expression for all four genes during liver stage development. As in *P. yoelii*, *P. berghei* *fabB/F* and *fabI* appear to be significantly transcribed already during the sporozoite stage.

vi) Genes encoding merozoite surface proteins (Fig. S8)

Merozoite surface protein 1 (MSP1) has been detected by immunofluorescence analysis from mid to late liver stages and similar levels of MSP1 expression was detected in blood stage and liver stage merozoites [18]. The RNA-seq data confirm this expression pattern, in that MSP1 expression is increasing towards late liver stage parasites. In early blood stages, MSP1 expression is high but then drops sharply in trophozoites to increase again in blood stage schizonts when merozoites are formed. The other MSPs have in common to be upregulated towards late liver stage and blood stage schizonts and except MSP8 that drops in expression at blood trophozoite stage.

vii) Selected genes for promoter driven reporter proteins (Fig. S9)

Heat shock protein 70 (hsp70) is highly expressed in all life cycle stages and the hsp70 promoter has been used to constitutively express reporter proteins in *P. berghei* [19,20]. In fact, it is considered superior to the eef1α promoter that was thought to be constitutively active and has already been used to generate *P. berghei* reporter cell lines [21–24]. Our RNA-seq data confirm the expected profile of both hsp70 and eef1α. However, whereas eef1α was found hardly expressed in sporozoites, hsp70 shows a more evenly expression throughout the life cycle and thus is better suited for the generation of reporter parasite lines.

**Supplemental Figures**

**
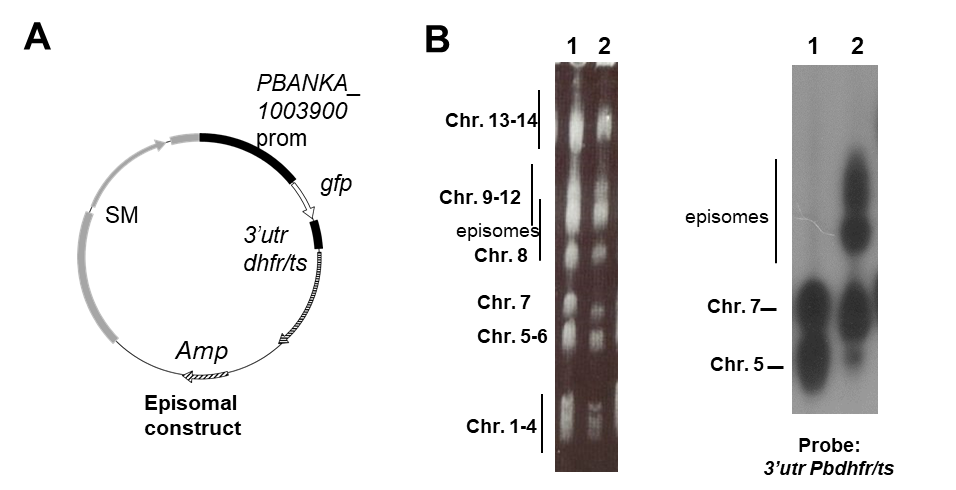
**

**Fig. S1** Generation and genotyping of parasites expressing *gfp* under control of the promoter of PBANKA_1003900 (PBANKA_1003900^GFP^). (**A**) Schematic representation of the plasmid used to generate parasites expressing *gfp* under control of the PBANKA_1003900 promoter. The construct contains the *Toxoplasma gondii* dihydrofolate reductase -thymidylate synthase (TgDHFR-TS) selectable marker cassette (SM: grey boxes and arrow) and the *gfp* expression cassette under control of the 1,7 kb of the PBANKA_1 003900 promoter region (black boxes and white arrow). (**B**) Southern analysis of pulsed field gel-separated chromosomes confirmed episomal transfection of construct in line 300 (lane 2). Right panel: chromosomes separated by pulsed field gel electrophoresis (FIGE) of two *P. berghei* mutant lines (line 300 and control line 299). Chromosomes are visualized by ethidium bromide staining of the gels. Left panel: separated chromosomes were hybridized with 3’utr Pb*dhfr/ts* recognizing the selectable marker and the GFP-expression cassettes of the introduced episomes and the 3’*utr* of the endogenous *Pbdhfr/ts* gene on chromosome 7. Lane 2 (exp 300) shows presence of episomal copies of the plasmid expressing GFP under control of the PBANKA_1003900 promoter and lane 1 show hybridization pattern of control line 299 with a construct integrated in chromosome 5.

**
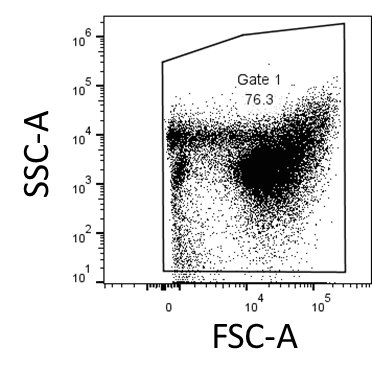
**

**A**

**B**

**
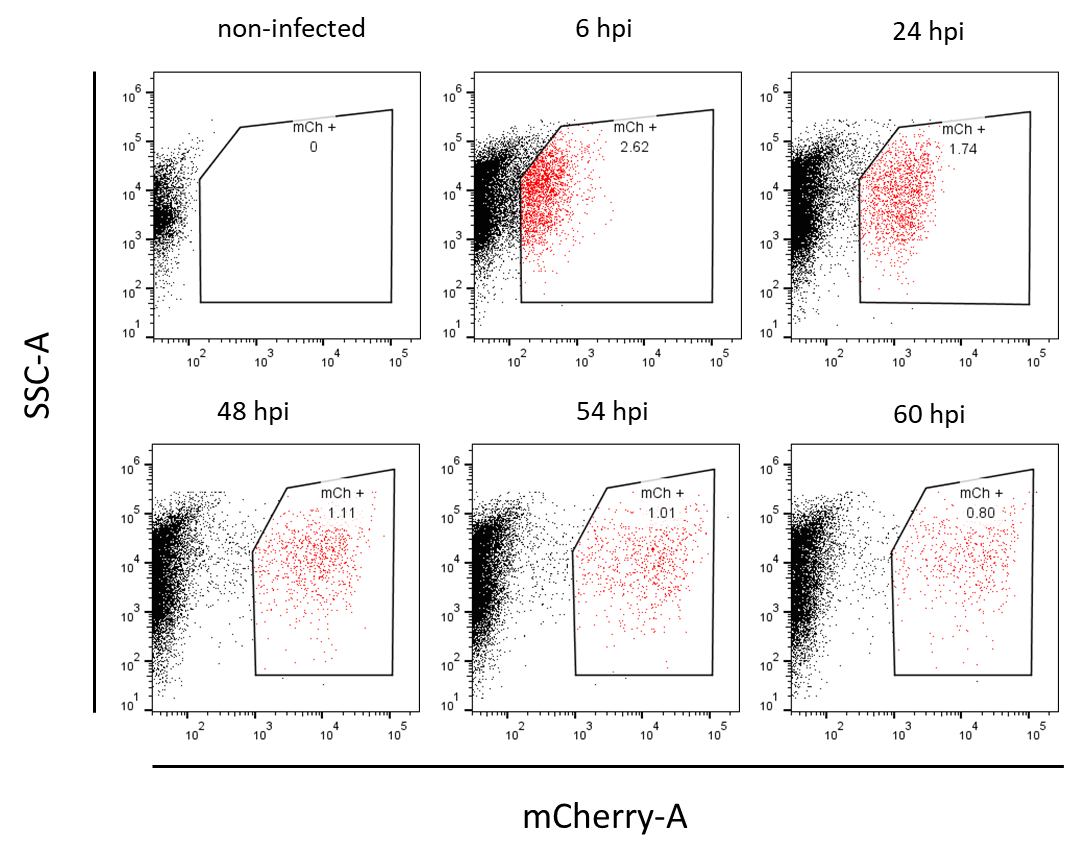
**

**Fig. S2** Fluorescence-activated cell sorting of PbmCherry_hsp70_ infected HeLa cells preserved in RNAlater. (**A**) Representative plot for FSC-A vs. SSC-A (**B**) Plots of sample set EEF_x_B (see Table S1) for SSC-A vs. mCherry-A (of Gate 1 selected events from A). mCh+ gated populations were sorted and processed for RNA isolation. The non-infected cells served as gating control. With increasing time of infection the gates were moved towards increasing mCherry-A signal in order to omit underdeveloped parasites (the numbers in the gates represent the percentage proportion of sorted cells of Gate 1 events)


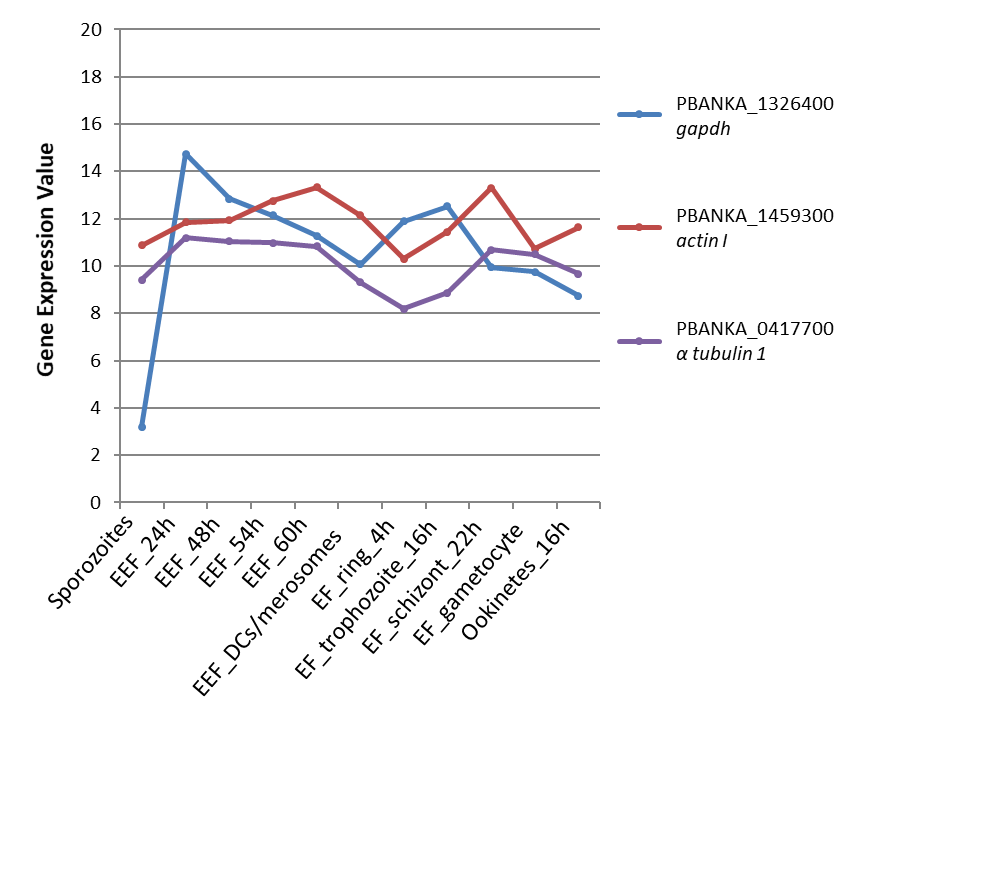
**Fig. S3** RNA expression profiles of 3 housekeeping genes (*gapdh, actinI, tubulin1*). Gene expression values corresponding to normalized and log2(x+1)-transformed read counts. The data were normalized with DESeq2 (with default parameters)[25].

**
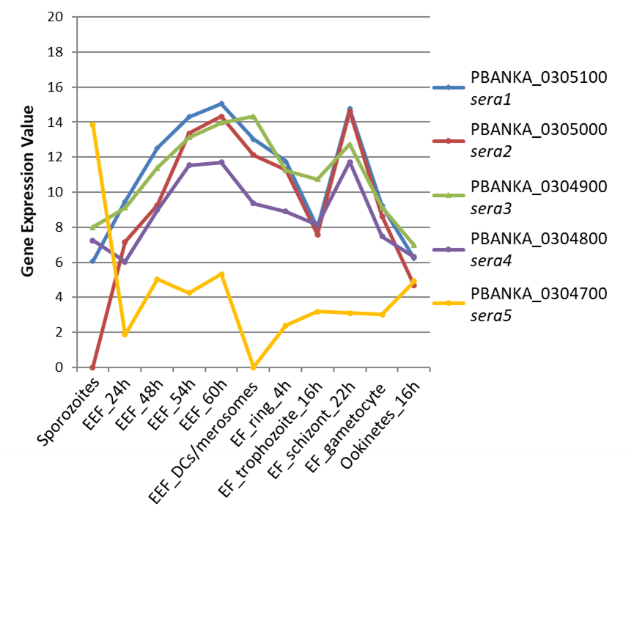
Fig. S4** RNA expression profiles of 5 genes encoding serine-repeat antigens, serine-type proteases (SERA1-5): Gene expression values corresponding to normalized and log2(x+1)-transformed read counts. The data were normalized with DESeq2 (with default parameters)[25].


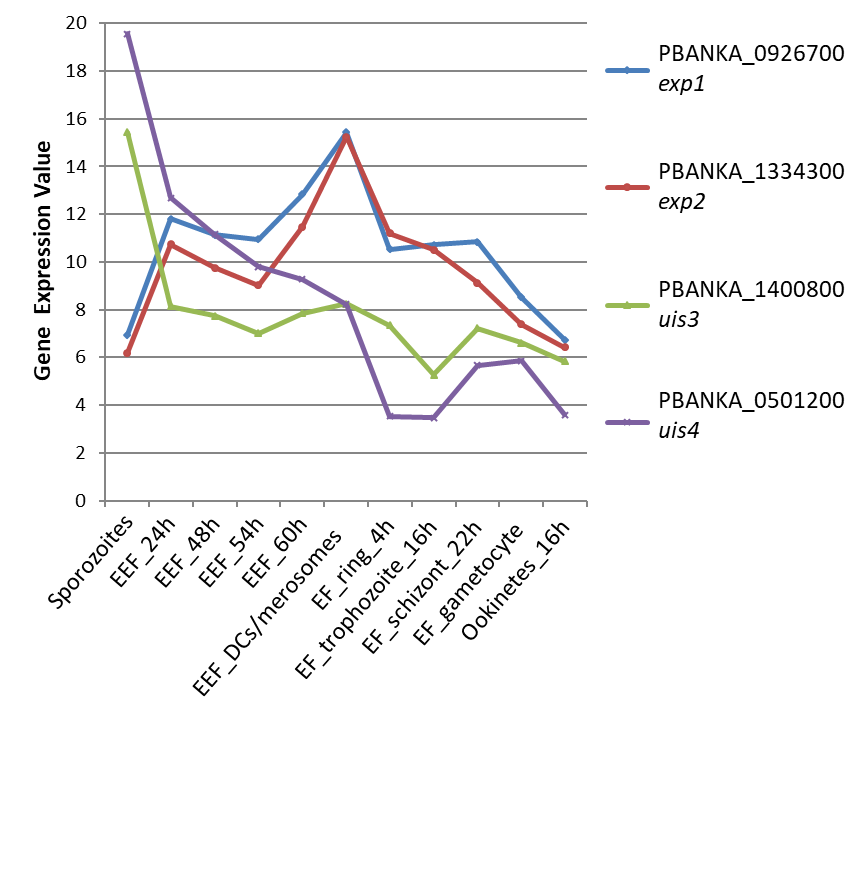
**Fig. S5** RNA expression profiles of 5 genes encoding proteins of the parasitophorous vacuole membrane (Exported protein 1, Exported protein 2, UIS3, UIS4). Gene expression values corresponding to normalized and log2(x+1)-transformed read counts. The data were normalized with DESeq2 (with default parameters)[25].

**
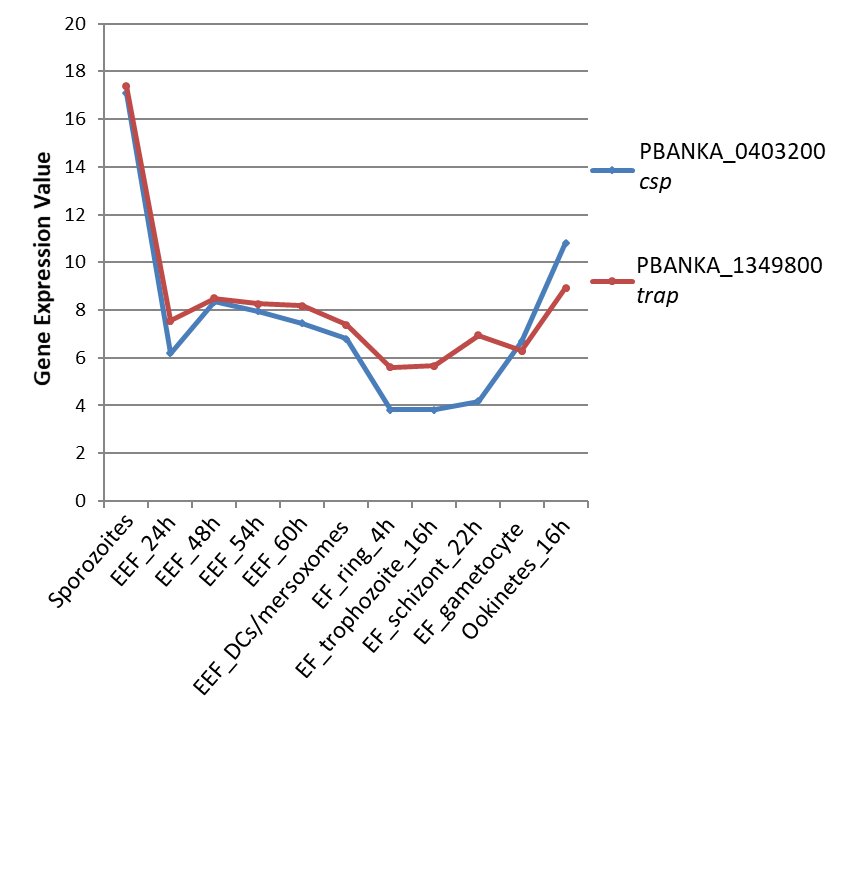
Fig. S6** RNA expression profiles of genes encoding 2 sporozoite surface proteins (CSP and TRAP). Gene expression values corresponding to normalized and log2(x+1)-transformed read counts. The data were normalized with DESeq2 (with default parameters)[25].


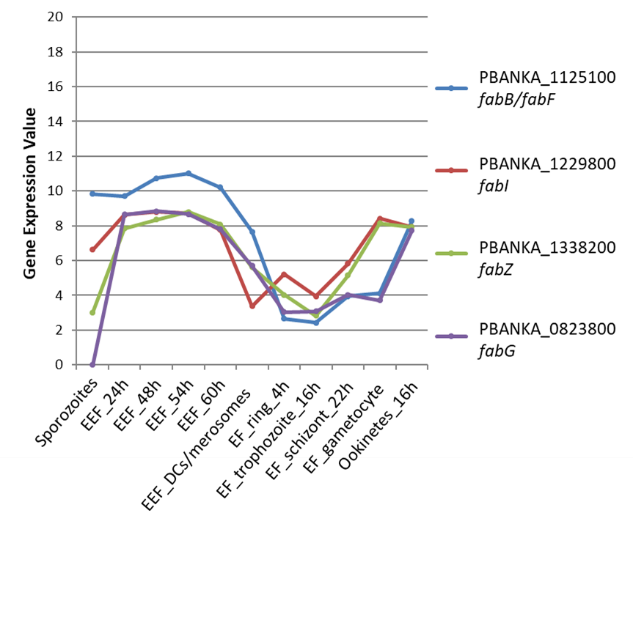
**Fig. S7** RNA expression profiles of 4 genes encoding enzymes involved in fatty acid biosynthesis (FabB/F, FabI, FabZ, FabG). Gene expression values corresponding to normalized and log2(x+1)-transformed read counts. The data were normalized with DESeq2 (with default parameters)[25].


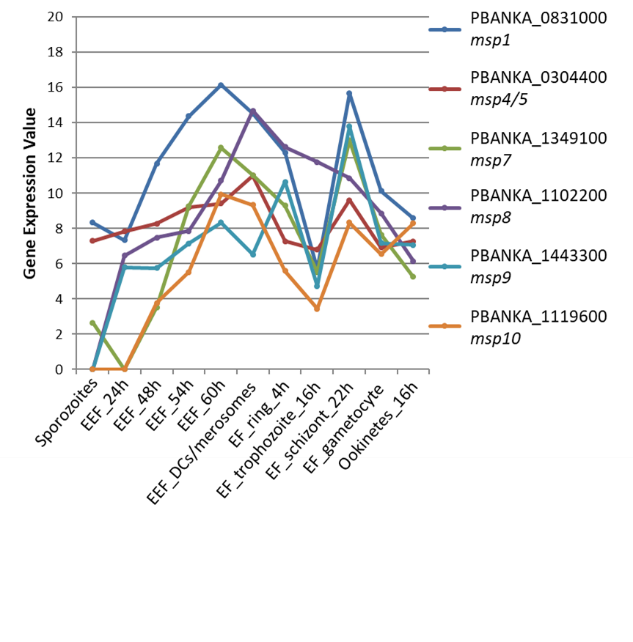


**Fig. S8** RNA expression profiles of 6 genes encoding merozoite surface proteins (MSP). Gene expression values corresponding to normalized and log2(x+1)-transformed read counts. The data were normalized with DESeq2 (with default parameters)[25].

**
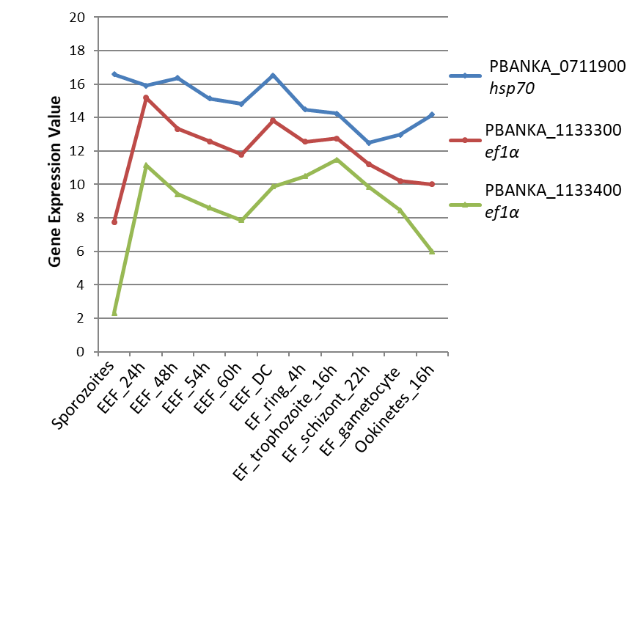
Fig. S9** RNA expression profiles of 3 genes whose promoter regions have been used to drive expression of fluorescent/luminescent reporter proteins (HSP70, two genes for EF1α). Gene expression values corresponding to normalized and log2(x+1)-transformed read counts. The data were normalized with DESeq2 (with default parameters)[25].

1.

hang Z, Jiang N, Zhang Y, Lu H, Yin J, Wahlgren M, et al. The TatD-like DNase of Plasmodium is a virulence factor and a potential malaria vaccine candidate. Nat Commun. 2016;7:11537.

2. Cha S-J, Kim M-S, Pandey A, Jacobs-Lorena M. Identification of GAPDH on the surface of Plasmodium sporozoites as a new candidate for targeting malaria liver invasion. J Exp Med. 2016;213:2099–112.

3. Cha S-J, McLean KJ, Jacobs-Lorena M. Identification of *Plasmodium* GAPDH epitopes for generation of antibodies that inhibit malaria infection. Life Sci Alliance. 2018;1:e201800111.

4. Schmidt-Christensen A, Sturm A, Horstmann S, Heussler VT. Expression and processing of Plasmodium berghei SERA3 during liver stages. Cell Microbiol. 2008;10:1723–34.

5. Helm S, Lehmann C, Nagel A, Stanway RR, Horstmann S, Llinas M, et al. Identification and characterization of a liver stage-specific promoter region of the malaria parasite Plasmodium. PLoS One. 2010;5:e13653.

6. Aly ASI, Matuschewski K. A malarial cysteine protease is necessary for Plasmodium sporozoite egress from oocysts. J Exp Med. 2005;202:225–30.

7. Putrianti ED, Schmidt-Christensen A, Arnold I, Heussler VT, Matuschewski K, Silvie O. The Plasmodium serine-type SERA proteases display distinct expression patterns and non-essential in vivo roles during life cycle progression of the malaria parasite. Cell Microbiol. 2010;12:725–39.

8. Tan TMC, Goh KL, Binh LN, Ting RCY, Kara UAK. Identification of a Plasmodium berghei antigen sharing common features with P. falciparum and P. chabaudi parasitophorous-vacuole membrane antigens. Parasitol Res. 1996;82:130–5.

9. Sturm A, Amino R, van de Sand C, Regen T, Retzlaff S, Rennenberg A, et al. Manipulation of host hepatocytes by the malaria parasite for delivery into liver sinusoids. Science. 2006;313:1287–90.

10. Sá e Cunha C, Nyboer B, Heiss K, Sanches-Vaz M, Fontinha D, Wiedtke E, et al. *Plasmodium berghei* EXP-1 interacts with host Apolipoprotein H during *Plasmodium* liver-stage development. Proc Natl Acad Sci. 2017;114:E1138–47.

11. Kaiser K, Matuschewski K, Camargo N, Ross J, Kappe SHI. Differential transcriptome profiling identifies Plasmodium genes encoding pre-erythrocytic stage-specific proteins. Mol Microbiol. 2004;51:1221–32.

12. Matuschewski K, Ross J, Brown SM, Kaiser K, Nussenzweig V, Kappe SHI. Infectivity-associated Changes in the Transcriptional Repertoire of the Malaria Parasite Sporozoite Stage. J Biol Chem. 2002;277:41948–53.

13. Silvie O, Briquet S, Müller K, Manzoni G, Matuschewski K. Post-transcriptional silencing of *UIS4* in *P* *lasmodium berghei* sporozoites is important for host switch. Mol Microbiol. 2014;91:1200–13.

14. Yoshida N, Potocnjak P, Nussenzweig V, Nussenzweig RS. Biosynthesis of Pb44, the protective antigen of sporozoites of Plasmodium berghei. J Exp Med. 1981;154:1225–36.

15. Sultan AA, Thathy V, Frevert U, Robson KJ, Crisanti A, Nussenzweig V, et al. TRAP is necessary for gliding motility and infectivity of plasmodium sporozoites. Cell. 1997;90:511–22.

16. Yu M, Kumar TRS, Nkrumah LJ, Coppi A, Retzlaff S, Li CD, et al. The fatty acid biosynthesis enzyme FabI plays a key role in the development of liver-stage malarial parasites. Cell Host Microbe. 2008;4:567–78.

17. Vaughan AM, O’Neill MT, Tarun AS, Camargo N, Phuong TM, Aly ASI, et al. Type II fatty acid synthesis is essential only for malaria parasite late liver stage development. Cell Microbiol. 2009;11:506–20.

18. Suhrbier A, Sinden RE, Nicholas J, Wiser MF, Holder AA. Expression of the Precursor of the Major Merozoite Surface Antigens During the Hepatic Stage of Malaria. Am J Trop Med Hyg. 1989;40:351–5.

19. Hliscs M, Nahar C, Frischknecht F, Matuschewski K. Expression Profiling of Plasmodium berghei HSP70 Genes for Generation of Bright Red Fluorescent Parasites. PLoS One. 2013;8:e72771.

20. Burda P-C, Roelli MA, Schaffner M, Khan SM, Janse CJ, Heussler VT. A Plasmodium phospholipase is involved in disruption of the liver stage parasitophorous vacuole membrane. PLoS Pathog. 2015;11:e1004760.

21. Billker O, Dechamps S, Tewari R, Wenig G, Franke-Fayard B, Brinkmann V. Calcium and a calcium-dependent protein kinase regulate gamete formation and mosquito transmission in a malaria parasite. Cell. 2004;117:503–14.

22. Franke-Fayard B, Trueman H, Ramesar J, Mendoza J, van der Keur M, van der Linden R, et al. A Plasmodium berghei reference line that constitutively expresses GFP at a high level throughout the complete life cycle. Mol Biochem Parasitol. 2004;137:23–33.

23. Janse CJ, Ramesar J, Waters AP. High-efficiency transfection and drug selection of genetically transformed blood stages of the rodent malaria parasite Plasmodium berghei. Nature Protocols. 2006;1:346–56.

24. Niz M De, Ullrich A-K, Heiber A, Soares AB, Pick C, Lyck R, et al. The machinery underlying malaria parasite virulence is conserved between rodent and human malaria parasites. Nat Commun. 2016;7:11659.

25. Love MI, Huber W, Anders S. Moderated estimation of fold change and dispersion for RNA-seq data with DESeq2. Genome Biol. 2014;15:55
